# Empowering Children with ASD and Their Parents: Design of a Serious Game for Anxiety and Stress Reduction †

**DOI:** 10.3390/s20040966

**Published:** 2020-02-11

**Authors:** Stéphanie Carlier, Sara Van der Paelt, Femke Ongenae, Femke De Backere, Filip De Turck

**Affiliations:** 1 IDLab, iGent Tower—Department of Information Technology, Ghent University—imec, Technologiepark-Zwijnaarde 126, B-9052 Ghent, Belgium; 2 Department of Experimental Clinical and Health Psychology, Ghent University, Henri Dunantlaan 2, B-9000 Ghent, Belgium

**Keywords:** Autism Spectrum Disorder, serious game, anxiety, stress, user empowerment, gamification, human-computer interaction, relaxation techniques, pervasive health

## Abstract

Autism Spectrum Disorder (ASD) is characterized by social interaction difficulties and communication difficulties. Moreover, children with ASD often suffer from other co-morbidities, such as anxiety and depression. Finding appropriate treatment can be difficult as symptoms of ASD and co-morbidities often overlap. Due to these challenges, parents of children with ASD often suffer from higher levels of stress. This research aims to investigate the feasibility of empowering children with ASD and their parents through the use of a serious game to reduce stress and anxiety and a supporting parent application. The New Horizon game and the SpaceControl application were developed together with therapists and according to guidelines for e-health patient empowerment. The game incorporates two mini-games with relaxation techniques. The performance of the game was analyzed and usability studies with three families were conducted. Parents and children were asked to fill in the Spence’s Children Anxiety Scale (SCAS) and Spence Children Anxiety Scale-Parents (SCAS-P) anxiety scale. The game shows potential for stress and anxiety reduction in children with ASD.

## 1. Introduction

Caring for a child with a developmental disability contributes to higher levels of parental stress than caring for a child with typical development, due to the challenging behavior of the children, judgment of others and a feeling of lack of support by their environment and the impact of ongoing stress on the family [1]. Moreover, caring for a child with Autism Spectrum Disorder (ASD) is associated with higher levels of parental stress than parenting children with a physical disability or children with developmental delays without ASD [2].

ASD is characterized by social interaction difficulties, communication problems and restrictive and repetitive behavior, according to the Diagnostic and Statistical Manual of Mental Disorders, Fifth Edition (DSM-V) [3]. The symptoms and their severity vary widely from children without intellectual disability and mild symptoms to children with intellectual disability and a lack of spoken language. ASD is a heterogeneous condition: the profile of each child or adult with ASD is unique [4]. An estimated 1 in 132 individuals suffers from ASD [5]. As a result of these challenges, related to social functioning and communication, people with ASD often suffer from other conditions, such as mood swings and anxiety. It is estimated that up to 72% of children with ASD suffer from co-morbidities [6], however, co-morbidity rates remain largely unknown for ASD, as co-morbid conditions can be difficult to diagnose [7,8,9,10]. Depression and anxiety are two of the most common ASD co-morbidities with rates around 40 to 50% [11].

Due to communication difficulties, children with ASD are often unable to express themselves when stressed or frustrated. Combined with unique symptoms in every child and overlap in symptoms of co-morbidities, it is challenging to find the appropriate response. As a result, temper tantrums are common, which can be an important cause of parental stress [1]. Determining the correct diagnosis or finding appropriate treatment is difficult due to the challenges in communication and uniqueness in symptoms [7].

Empowering both the children and their parents by raising awareness of the child’s anxiety and stress can result in better management of the child’s temper tantrums, which can result in a decrease in stress and anxiety. The term “patient empowerment” is used in many different settings and domains, but no general definition exists. The consensus is that patient empowerment is used to describe situations where patients and users are encouraged to take control of their health management [12]. In other words, patient empowerment is enabling patients to actively understand their health [13], but also the support to restore a sense of hope, respect, self-efficacy and the drive to face seemingly insurmountable challenges [14].

The main objective of the study is to design a proof of concept for a serious game for children with ASD to reduce stress and anxiety and to evaluate the feasibility of designing technologies for the empowerment of children with ASD and their parents. The main hypotheses of this study were

1. if the child indicates reduces levels of stress and/or anxiety after a gaming session,
2. if the game is adopted as a tool for stress and anxiety reduction and is used spontaneously,
3. the supporting application provides insight into the child’s anxious feelings and when they occur.

Serious games have a purpose other than pure entertainment, such as education, skill enhancement or behavioral changes [15,16,17]. Serious games are increasingly more popular for use in the health care sector and have shown to be beneficial for the mental health and well-being of children and adults [18,19]. Serious games exist for the treatment of depression, post-traumatic stress disorder, autism spectrum disorder, attention deficit disorder, cognitive functioning and alcohol use disorder [16,18,20].

As a result of the communication and social interaction difficulties, characteristic for ASD, verbal, face-to-face therapies might lead to an increase in anxiety and stress in children with ASD. Additionally, the real world contains many confusing multi-sensory distractions, often anxiety-inducing and can increase problems with social communication. A computer-based environment, such as a serious game could provide a safe environment for practicing newly acquired skills to avoid these anxiety-inducing situations [21,22,23]. Moreover, it is often reported that children with ASD have a predilection for technology and prefer video games over social interaction [21,24].

During the course of this research, a close collaboration with specialized therapists and their centers was set-up. During each step of this process, the therapists were consulted for guidance and feedback. After a thorough literature search and combined with the expertise of the therapists, guidelines for the design and implementation of a serious game for children with ASD were formed. Based on these guidelines, a prototype of a serious game and a supporting parent app were implemented, which were both tested by three families. These usability tests were conducted to evaluate if the proposed guidelines and resulting prototype fit the needs of children with ASD. Furthermore, it was evaluated that if the basic parental application can be a starting point for increased parental empowerment.

The object of this first user study was to test the feasibility of the prototype of the game and the study design. This paper is an extension of a shorter conference paper [25]. Whereas the original conference paper focuses on reporting the first preliminary results of the user study with three families, this extended paper elaborates on the entire software design process of a serious game for children with ASD in co-creation with specialized therapists from concept to prototype. This paper reports on all the requirements and difficulties that need to be considered throughout the process of designing and evaluating a serious for this specific target audience, from defining the non-functional requirements, over the architecture to an extensive analysis of the incorporated game elements that contribute to learning in serious games. Moreover, this paper examines if the entire design process satisfies the guidelines for patient empowerment in e-health applications. The findings reported in this paper will be incorporated into a future follow-up study to further validate the preliminary results of the first user study.

First, in Section 2, stress, and anxiety in children with ASD will be discussed. Next, relevant related work will be presented in Section 3, followed by an introduction to the New Horizon game and SpaceControl application in Section 4. An interdisciplinary approach was chosen for the design and implementation of both applications, which will be discussed in Section 5. Next, the non-functional requirements and the architecture are examined in detail in Section 6 and Section 7 respectively. Important technological choices and implementation decisions are reported in Section 7.3 and Section 8. Finally, the evaluation set-up and its results are discussed in Section 9 and Section 10.

## 2. Stress and Anxiety in Children with ASD

Anxiety disorders are among the most common psychiatric conditions in children and adolescents, with estimated rates of 6.5% in 6- to 18-year-olds [26]. Severe stress or anxiety can disturb daily life, school performance or relationships with peers and family. Some of the most common anxiety disorders in children with ASD are specific phobia, social phobia, obsessive-compulsive disorder (OCD) and generalized anxiety disorder (GAD).

To decide on the correct intervention for the treatment of stress and anxiety, it is important to investigate when stress and anxiety appear. The measurement of stress and anxiety is, however, no easy feat. Stress and anxiety can be detected and measured using subjective or objective measures. Subjective self-report measures, often questionnaires or scales, indicate the individual’s perceived stress level, while biomarkers give an objective measure of a person’s state. Biomarkers can be monitored by smartphones and wearables for real-time stress and anxiety detection, however, existing technology has yet to be perfected [27].

Treatment of an anxiety disorder depends on the type of disorder, the severity of the complaints and the presence of co-morbidity and can be pharmacological or include psychotherapy. One of the most used forms of therapy for anxiety treatment is Cognitive Behavioural Therapy (CBT) [28], whereas stress management is recognized as an effective treatment to reduce stress, including different relaxation techniques [29].

The following paragraphs will first, in Section 2.1, discuss Cognitive Behavioural Therapy (CBT) and relaxation techniques for the treatment of anxiety and stress disorders, followed by Section 2.2, where anxiety and stress in children with ASD will be discussed, listing possible modifications of CBT for the treatment of children with ASD.

### 2.1. Cognitive Behavioural Therapy (CBT) and Relaxation Techniques

The most used form of therapy for the treatment of anxiety disorders is Cognitive Behavioural Therapy (CBT) [28].

CBT aims to identify and challenge anxious thoughts and beliefs and change problematic behavior. It uses behavioral techniques, such as exposure and relaxation, as well as cognitive techniques [11]. Stress management is recognized as an effective treatment to reduce stress and includes techniques such as Progressive Muscle Relaxation (PMR) or mental imagery. Relaxation and stress management techniques are often used to cope with negative automatic thoughts and emotions, which makes it an important element of CBT [30]. The most relevant relaxation techniques proven to be effective in reducing stress and anxiety symptoms are:

- Focused breathing: breathing exercises are considered fundamental for mental and physical well-being. Inhaling alerts our body, while exhaling has a calming effect [31]. By focusing on breathing and diaphragm movement, the nervous system can be influenced and the heart rate can be slowed down [31].
- Guided imagery (GI): visualization uses personalized images, symbolic health problems and an audio or written script to promote health. Focus is on linking elements of relaxation in the image to the physiologically relaxed state simultaneously being experienced by the patient, using all sorts of sensory information [32].
- Biofeedback: a process to learn how to change physiological activity to improve health and performance. The presentation of physiological signals, e.g., heart rate and skin temperature, and changes in thinking or behavior, teach the patient to alter physiological activity [32].

### 2.2. Modifications for ASD

Research suggests that anxiety disorders are substantially more common among children with ASD compared to their typically developing peers. Settipani et al. report rates of approximately 42. It is not well understood if the same struggles exist for anxiety disorders in youth with ASD as in their peers without ASD [8]. Children with ASD suffering from anxiety disorders might face additional anxiety-related issues related to more dysfunctional behavior and general internalizing problems: more aggression and irritability, an increase in self-injury in lower functioning youth, more negative automatic thoughts, self-reported loneliness and depression symptoms [8]. Poorer communication skills and increased social withdrawal are common symptoms of anxiety disorders, but research shows that children with ASD may report more anxiety if they have stronger communication skills [8].

There are some limitations to the use of CBT for anxiety in children with ASD. First, CBT should focus more on a visual, concrete approach, instead of verbal face-to-face sessions. Verbal therapy sessions can be challenging for children with ASD, who are often cognitively and socially impaired, making it difficult to learn the necessary skills, which can negatively influence their motivation [19,24]. Second, frequent practice and exposure are necessary, as they have trouble with the generalization of learning, i.e., applying what they have learned to daily situations [19,24]. Third, therapy might be limited available or accessible, due to long waiting lists or treatment hours that might fall during work or school hours [19,24,33]. Finally, the cost-effectiveness of mental health treatment can be a barrier for some: some families cannot afford treatment or schools lack the funds to offer the appropriate treatment [19,24].

Limited research exists on the treatment of co-morbid anxiety disorders in children with ASD, but the consensus is that CBT, with certain modifications, can be used as a treatment for anxiety [11]. The National Institute for Health and Care Excellence (NICE) and a few other reviews list some guidelines in the modification of CBT for children with ASD regarding structure and content, which will be briefly summarized in Table 1 [11,34,35]:

## 3. Related Work

The following paragraphs will discuss related work, relevant for the design of a serious game and supporting parental application for children with ASD.

To ensure that both applications can empower both parents and children, the guidelines for developing e-health application for patient empowerment, discussed in Section 3.1, were taken into account from the start to decide on the necessary steps during the design process. Information about how serious games can attribute to learning is summarized in Section 3.2. Section 3.3 provides an overview of the limitations of existing technologies for empowering parents of children with ASD and lists existing serious games for anxiety reduction. Finally, Section 3.4 summarizes guidelines found in the literature for the design of software for children with ASD.

### 3.1. Patient Empowerment through E-Health

Patient empowerment often focuses, in the first place, on informing the patient about his/her illness. However, long-term illness or chronic diseases can have a significant influence on the patient’s mental health and quality of life (QOL). For example, for pediatric cancer patients, patient and parental stress are omnipresent, e.g., prolonged hospitalization, chemotherapy, surgery [14], resulting in a decrease in QOL and increased anxiety. Often, these stressors are uncontrollable, resulting in the need to adjust one’s perception or reaction of the stressful situation instead of being able to change the situation itself [14]. Patient empowerment can address patient depression and anxiety by learning the patient to understand and positively influence his/her health by engaging them in their health management [14].

E-health and mobile health can be used to empower patients in an affordable and accessible manner, however, these applications are not always designed according to the patient empowerment requirements [12]. E-health applications are often designed to reduce the demand for care for patients and create a more efficient work process for the healthcare professional as patients are encouraged to self-manage some activities supported by the e-health application [12]. Alpay et al. [12] lists requirements for the empowerment of patient with e-health in four areas:

- Insight into own health condition: access to medical data can help the patient to comprehend their health situation better and manage it more carefully.
- Making informed choices: the patient can make an informed decision on how to manage his/her condition if he/she is aware of the current health status and the options that can be taken.
- Self-care activities and habits: the self-care options the patient has chosen can result in daily health-related goals and activities while maintaining a good QOL.
- Living independently: living in your environment contributes to empowerment, as it is a safe and familiar environment.

Alpay et al. [12] summarizes guidelines and best practices from reviewed studies for developing e-health applications for patient empowerment, visible in Table 2.

### 3.2. Game Elements of Serious Games

A serious game is a game that is designed for a goal other than pure entertainment, such as education, skill enhancement or behavioral changes [15,16,17]. No generally agreed-upon definition of what constitutes a serious game exists, however, some agreement is found in defining the goal of a serious game to be to educate or train as well as entertain the user [15].

Serious games are widely used in many different application domains without explicit knowledge as to why they are effective teaching tools [36,37]. It is not clear which game elements lead to which learning outcomes. Defining these game attributes is not an easy task, plenty of different game attributes and definitions exist in the literature, but they often overlap [36]. Bedwell et al. [36] reorganized the 19 attributes relevant to learning, according to Wilson et al. [37] into nine categories: action language, assessment, conflict/challenge, control, environment, game fiction, human interaction, immersion, rules and/or goals. The different game elements and their respective game attributes can be found in Table 3 [15,36,37,38]:

### 3.3. Serious Games for Mental Health

The main application domains of serious games remain education and health care [39]. Serious games are increasingly used for mental health care treatment: serious games exist for the treatment of depression, post-traumatic stress disorder, ASD, attention deficit hyperactivity disorder (ADHD), cognitive functioning and alcohol use disorder [16,20]. Examples of serious games that are used for mental health care treatment are Playmancer, a biofeedback game for impulse-related disorders [40], The Journey to Wild Divine, a biofeedback stress management game for children with AD/HD [41]. Botanical Nerves is a serious game for stress management using biofeedback [42] and the Mindlight game uses neurofeedback to reduce stress and anxiety [43]. Serious games for mental health care treatment often incorporate biofeedback in the game, requiring the use of wearables. However, real-time stress and anxiety detection using wearables is no easy feat as existing technology has yet to be perfected [27]. Moreover, incorporating such wearables in the game could provide an extra obstacle for children with ASD as they have to be worn correctly and can be uncomfortable to wear.

For children with ASD, a computer-based environment can reduce the confusing multi-sensory distractions of the real world, which are often anxiety-inducing and can increase problems with social interactions. Such a digital environment provides a safe environment for the child in which he/she can practice newly acquired skills [21,22,23].

Several technology interventions for ASD exist, including computer programs, virtual reality and robotics [44,45,46,47,48]. Innovations in the technology used for children with ASD focus mainly on social interaction difficulties, more specifically, emotion recognition or language skills, however, only a handful incorporate serious games [21,49,50,51,52].

Only two studies were found that discuss stress and anxiety reduction in children with ASD using a game intervention:

- Mindlight: Mindlight is a neurofeedback game developed to reduce and prevent anxiety in typically developing children [43]. The game uses CBT techniques but focuses on training children how to cope with anxiety instead of explaining it. Therefore, the game needs to trigger these feelings of anxiety so the player can learn how to regain their calm to progress in the game. The study showed that playing Mindlight significantly reduced anxiety symptoms. Wijnhoven et al. [24] hypothesized that Mindlight has the potential to reduce anxiety symptoms in children with ASD, since a serious game can reduce some of the previously mentioned limitations of CBT. At the moment of writing, no results were yet reported [24]. The Mindlight game is not freely available so it could not be used for comparative testing.
- ReacTickles^©^: the ReackTickles software aims to provide relaxation, encourage spontaneous play and support learning for children with ASD by encouraging exploration and experimentation. It tries to recreate the feeling of repetition using the sensory qualities of gravity, temperature, chance, space, and enclosure to encourage tapping, smoothing, circling using an interactive whiteboard [23]. The ReacTickles software is more interactive and sensory play space for children than an actual game and it allows children to focus on the effect of their actions instead of completing a sequence of steps to perform a specific task [23]. The ReacTickles platform focuses on children with severely delayed developmental ages, around 1 to 2 years old, and has thus not been incorporated for comparative testing.

In addition to a lack of serious games for anxiety and stress reduction, a limited amount of interventions exist for the empowerment of parents of children with ASD that include both parents and children in the process. Most of the technology available for parents are platforms to coach the parents in areas where access to therapy is scarcely available [53,54,55]. These platforms train the parents in ASD intervention techniques which they can then apply to their children. Although these platforms increase knowledge on possible treatment techniques, they, however, do not provide the user with any information regarding the child’s development or feelings.

The following paragraph will discuss some general guidelines for computer-based interventions for children with ASD.

### 3.4. Designing Technology for Children with ASD

Some studies list guidelines for the design of software or serious games for children with ASD, established from the experience gained from empirical studies, collaborations with therapeutic centres, interviews with parents and user observations [22,49,56,57]. The most relevant guidelines are discussed in Table 4.

The previous sections have shown that children with ASD and their parents are faced with numerous problems and co-morbidities, as a result of ASD. The manifestation of ASD symptoms is unique and it can be difficult to discern ASD symptoms from other co-morbidity symptoms as they often overlap. Therefore, finding the appropriate treatment can be challenging, even more so because of the typical communication difficulties that characterize ASD, as children with ASD are often unable to clearly express their feelings.

CBT has shown to be beneficial for children with ASD, but has some limitations. Serious games can provide a solution for these as they focus more on the visual aspect, reduce verbal face-to-face communication and are easily accessible. It was shown that serious games for ASD do indeed exist, but mostly focus on other ASD challenges, such as emotion recognition. Teaching children with ASD how to recognize emotions can be the first step towards emotion awareness but has little impact on empowering parents and children daily to reduce temper tantrums. Very few applications exist for empowering parents of children with ASD, whereas it was recommended, in Table 1, to modify CBT to include parent involvement as they are vital for continuing and applying the learned skills and behavior in a home environment.

## 4. New Horizon and SpaceControl: A Serious Game for Stress and Anxiety Reduction

Since anxiety and stress is such a common problem in children with ASD and only two studies, discussing an existing gameful intervention, were found, it was decided to design a serious game that integrates CBT techniques and takes the modifications and guidelines for ASD into account. As relaxation is such an important aspect of CBT, it was decided to incorporate two techniques into the game.

This research aims to investigate if a serious game can reduce stress and anxiety in children with ASD and how the needs of the target audience affect the design process and guidelines. Wearables are often used for serious games for mental health, however, these can provide an extra obstacle for young children with ASD. It was thus decided not to include biofeedback for the first phase of the game, so it could be evaluated how children receive and react to the game without any hindrances from wearables.

The following paragraphs will illustrate the process from design to proof of concept of a serious game for stress and anxiety reduction for children with ASD, more specifically, for the New Horizon game. The first paragraphs will elaborate on New Horizon and why it is suited for this research. Finally, the design of the relaxation module and the app for the parents, SpaceControl will be discussed.

### 4.1. What Is New Horizon?

**Figure 1:**
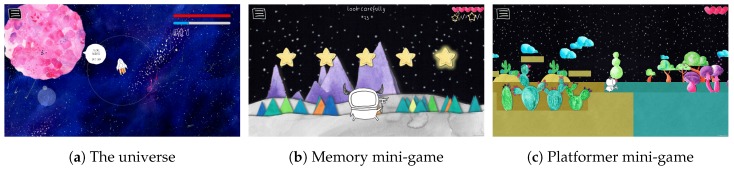
Screenshots from the New Horizon game. ©Stéphanie Carlier.

New Horizon is a 2D mobile exploration and puzzle game. The main game environment is an infinite universe with an infinite amount of discoverable planets. The main character, Jimmy, traverses this universe within his spaceship, as shown in Figure 1a, landing on planets to complete mini-games and earn experience. Two regular mini-games are designed, containing no CBT techniques:

- Memory mini-game: In this mini-game, several stars are visible. The stars will light up in a specific order. It is your mission to imitate this sequence exactly before time runs out. The memory mini-game is visible in Figure 1b.
- Platformer mini-game: In this mini-game, you need to explore unknown territory. To get back to your ship, you need to avoid danger and any possible hazards on your way. The faster you succeed, the more experience you will earn. The platformer mini-game is visible in Figure 1c.

**Figure 2:**
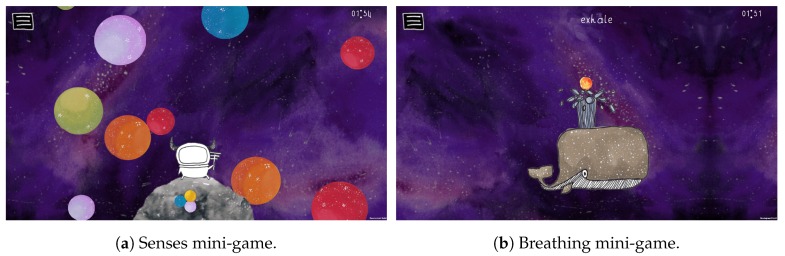
Screenshots from the relaxation module of the New Horizon game. ©Stéphanie Carlier.

Two mini-games were created that incorporate relaxation techniques, namely the visualizations or guided imagery relaxation technique and focused breathing, which was discussed in Section 2.1:

- Senses mini-game: the objective of the senses mini-game, visible in Figure 2a, is to pop as many bubbles of the correct color within the time limit, to collect the stardust inside the bubbles.
- Breathing mini-game: for the breathing mini-game, visible on Figure 2b, the objective is to focus on your breathing and inhale and exhale at the correct time to earn rewards, in the form of a snack for the space whale.

### 4.2. SpaceControl: A Tool for Parents

Caring for a child with ASD can lead to higher levels of stress for the parents due to the challenging behavior of the children, judgement of others, a feeling of lack of support by their environment and the impact on the family due to ongoing stress [1]. Due to the typical difficulties with communication, it is often hard for the parents to know how their children are feeling. Section 3.3 discussed the lack of digital health interventions for parent empowerment on a daily basis.

**Figure 3:**
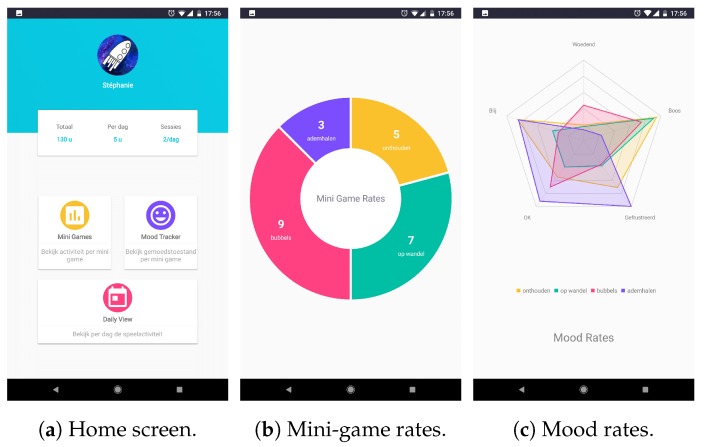

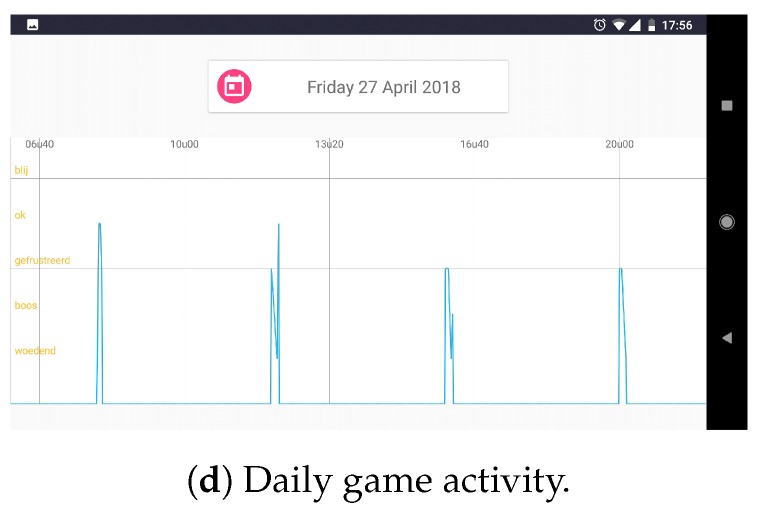
SpaceControl: activities.

The objective of the SpaceControl app is to empower the parents and to reduce the burden of dealing with daily temper tantrums. The app visualizes information about the gaming behavior of the child, intending to provide the parents with some insight into their children’s feelings. Information about the mood of the children can learn the user if the child often indicates the same mood after playing a specific type of mini-game or if he/she finds a certain type of mini-game enjoyable or not. Information about the number of times each mini-game has been played can give information about the favorite mini-game of the child but also indicate if the child is showing signs of obsessive behavior by repeatedly playing the same type of mini-game. By tracking the gaming sessions, the user can also get insight into the tendencies of the child of playing the game at, perhaps, fixed moments or when feeling down. Figure 3 gives an overview of the different graphs present in the SpaceControl application, Figure 3a displays the home screen with general information about total gaming duration and more, Figure 3b shows the mini-game rates, Figure 3c displays the mood rates and Figure 3d indicates the daily game activity.

By providing the children with an easy, communication-free option to express their mood, i.e., by simply selecting a smiley, the parents could gain more insight into the mood of their children and the factors that provoke these emotions.

### 4.3. Objective and Vision

As stated in Section 3.4, many children with ASD prefer the use of technology over social interaction and serious games have proven to be beneficial for the mental health of both children and adults. This section will discuss why New Horizon specifically could prove to be beneficial for the use of stress and anxiety reduction in children with ASD.

The symptoms of ASD and their severity vary wildly and are unique for each child. Whereas for some, music might be an ideal tool for reducing stress and anxiety, for others, it could trigger these feelings even more. Therefore, an individualized approach is often necessary. The structure of New Horizon, using mini-games in a larger whole, the universe, can provide this individualized approach: the choice of playing a specific type of mini-game is entirely up to the child and in no way necessary to be able to play other aspects of the game.

As stated in Section 2.2, CBT can be used as a form of therapy to reduce stress and anxiety in children with ASD, but it requires some modifications of its limitations. CBT requires face to face verbal interaction and can be cognitively complex with very little application to real-life situations. Furthermore, there can be a long waiting list for therapy, limiting exposure opportunities. The focus needs to shift from the verbal session to a more visual approach, providing sensory information. New Horizon is a mobile game and can thus be used in therapy, at home or at school, wherever and whenever needed. The short mini-games of several minutes can be played as a short intermezzo when stressed and anxious feelings are starting to rise. One can choose to play just one mini-game or multiple mini-games back to back if a longer session is called for. Secondly, a mini-game and its content are completely separable from the rest of the game, which lends itself to translating certain components of CBT into a mini-game, e.g., relaxation techniques. By translating these components to a mini-game, New Horizon can be used as a visual aid for certain aspects of CBT.

According to the expertise of the consulted therapists, the graphical aspect of New Horizon can be appealing for the targeted age category of 6 to 10 years. The children can look at Jimmy as a digital friend and the universe as a calming place to escape to when they are feeling scared or stressed. Since it is a game, the children can practice new relaxation techniques repeatedly in a safe gamified environment, without this having any effects in real life if they struggle to master the techniques at first.

## 5. Interdisciplinary Approach

The symptoms of ASD and their severity differ widely from child to child, making each child unique [4]. Therefore, technology designed for this target audience will be highly influenced by their specialized needs and requirements [22]. The guidelines found in literature, discussed in Section 3.4 can be used as a starting point for designing software for children with ASD, but remain general. To design a user-friendly game for children with ASD, expert knowledge about the children’s unique needs is necessary, requiring a more participatory design approach, including specialized therapists, familiar with the difficulties this disorder brings, in the design process [49].

This research has been established in cooperation with researchers from the Ghent University Faculty of Psychology and Educational Sciences. Based on their experience, two therapeutic centers were contacted for collaboration during the design process of the game.

**Figure 4:**
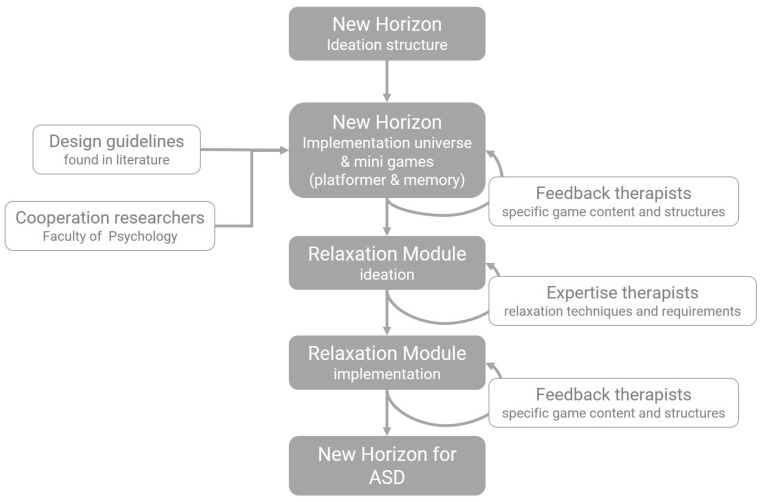
Design flow diagram.

The expertise of an occupational therapist, a behavioral therapist and a psychologist was called upon during the design and implementation of the game and the two relaxation mini-games. During this iterative process, shown in Figure 4, the therapists, together with other therapists of their centers, reviewed the game in search of necessary alterations and provided their ideas for implementing relaxation techniques in a gameful manner. These multiple sessions with the therapists resulted in feedback that needs to be taken into account for designing a game for children with ASD to reduce stress and anxiety. These remarks can be applied to any game, but the given examples apply to the New Horizon game specifically. The therapists emphasized that these remarks are not fixed rules, but merely guidelines, based on their experience with children with ASD. These guidelines were taken into account for the design of the game and are listed in Table 5.

## 6. Non-functional Requirements

When designing games and software for children with ASD, it is important to pay attention to the overall operation of the software. Quality attributes or non-functional requirements can influence the operation of the system and can, therefore, define or influence the architecture of the system.

Children with ASD often have a predilection for technology and games, but the guidelines, listed in Section 3.4, need to be taken into account. Guidelines that impact the design of the system are the need for repeatability and predictability in the game and the minimization of transitions and loading screens to avoid unwanted obsessive behaviour.

Based on these design guidelines, some important non-functional requirements, or quality attributes, can be defined that were taken into account during the design process of the game: usability, performance and modifiability. These quality attributes were taken into account during the entire design and implementation process:

- Usability: creating a usable tool for children with ASD is of utmost importance, since, more than in typically developing children, small or insignificant seeming elements, e.g., sound effects, can lead to unwanted and obsessive behavior and increased stress and anxiety. To ensure that the usability of the game has been tailored to the needs of children with ASD, the guidelines of Section 3.4 have been taken into account combined with the regular feedback and input of specialized therapists during the entire design process.
- Performance: performance is an important quality attribute to take into account as a less performant game can result in long waiting times between transitions of game states. Since the user is constantly seeking new mini-games to complete within the universe, the number of transition times from the universe to the planet and back can increase rapidly. Therefore, the transition time between these two game states is reduced to a minimum and does not include any animations or static images.
- Modifiability: a game developed for children with ASD should allow repetition to create a predictable environment for the child, nonetheless should there be room for evolution of the player. Modifiability should be taken into account, as creating new mini-games not only helps to keep the user interested in the game but creates new opportunities to create relaxation and incorporate CBT techniques. Therefore, adding new mini-games to the universe or changing existing mini-games should be easily possible.

## 7. Architecture

**Figure 5:**
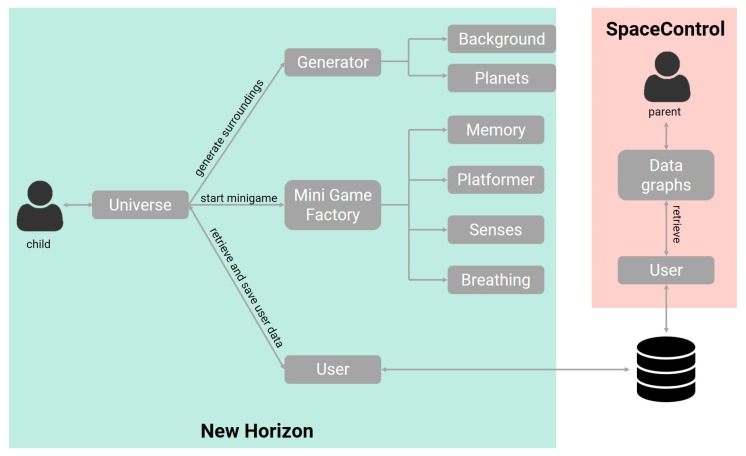
Overview of the different components.

This section will discuss the architecture of the New Horizon game and the SpaceControl application. Figure 5 shows an overview of the different components in the system and their interaction. First, in Section 7.1, the architecture of the New Horizon game with the different mini-games will be examined in detail, followed by Section 7.2, discussing the SpaceControl architecture. Section 7.3 will briefly discuss some technological choices made during the design process.

### 7.1. Architecture New Horizon

Figure 5 shows an overview of the different components of the New Horizon game. The universe component refers to the universe in the game, where the player can fly around in his spaceship, encounter planets, with or without hostile satellites protecting them. The universe component is responsible for the generation of the player’s close surroundings, the starting of a mini-game, and retrieving and storing user data. The universe generation takes place in real-time and is based on the player’s location. It consists of two phases, namely the generation of the background and the generation of the planets, more specifically, their locations and type of mini-game. To ensure the performance, one of the quality attributes, is high and the presence of transition waiting times and loading screens is minimized, only the close surroundings of the player are generated and not the entire universe to ensure that the generation can be done quickly.

Next, the universe component is responsible for storing and retrieving user data. User data refers to the current location of the player, the earned experience points, the unlocked spaceships but also the gathered data regarding the user’s gaming data, which will be discussed in more detail in Appendix A.3. This data is then stored in a database to be displayed by the SpaceControl application.

**Figure 6:**
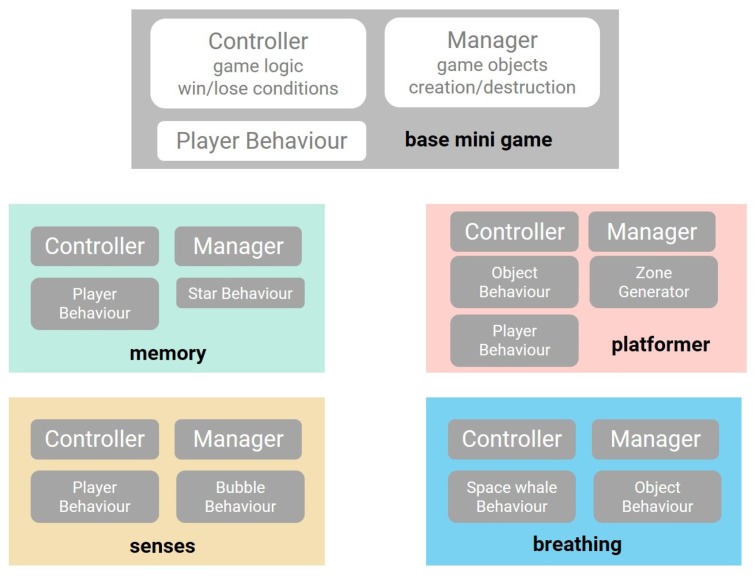
High level overview of the mini-game structure.

Finally, the universe is responsible for starting a mini-game on a planet. When the user lands his spaceship on a planet, the mini-game factory is responsible for loading the respective mini-game. Modifiability is an important quality attribute to ensure that adding new mini-game types can be achieved easily, without making modifications to other game components. Figure 6 shows an overview of the mini-game structure, emphasizing modifiability, as the structure of each mini-game is similar, containing a manager and a controller. Each mini-game needs to implement at least the controller and manager from the base mini-game. Nearly all mini-games will also feature a player behavior component, as well as other additional object-specific components. The managers are responsible for managing the Unity game objects of a specific mini-game. They are responsible for the creation and destruction of a mini-game. The controllers are responsible for the game logic and keeping track of win/lose conditions. Creating a new type of mini-game can be done by creating a controller and manager component that implement the base mini-game. If certain game objects are present in multiple types of mini-games, the existing object behavior components can easily be reused.

#### 7.1.1. Memory Mini-Game

In a memory mini-game, the goal is to replicate sequences without making mistakes. One mini-game consists of two or more sequences of a given length. If a player completes a sequence, the next sequence is shown. Once all sequences have been completed, the challenge is completed. A score is given based on how many mistakes were made and how much time was left. The player starts the challenge with a set number of chances, depending on difficulty, as well as with a countdown timer. Should the player run out of chances or the timer counts down to zero before all sequences have been completed, the challenge is lost.

The controller generates the star sequences based on the seed that was calculated based on the planet’s location within the universe.

#### 7.1.2. Platformer Mini-Game

During a platformer mini-game, the player has to try and reach the end of the level without dying. The level is filled with obstacles and hazards that will hinder the player’s progress. The reward the player gets is based on the time taken and the number of lives left. Should the player run out of lives before reaching the end of the level, the challenge is lost.

A level is generated by generating new zones, based on the seed, through the use of the Zone Generator. A zone is defined as a group of objects, sharing a specific theme. So far, two zones exist: the cactusscape and the forestscape. Each zone comes with its own particular hazards and obstacles. A zone, in its turn, consists of one or more “set pieces”, the smallest playable level element. The Zone Generator is able to smoothly place all set pieces next to each other on the correct height, creating a continuous, playable zone.

#### 7.1.3. Senses Mini-Game

Following the same structure, discussed in Section 7, the mini-game consists of a manager, which manages the Unity game objects and a controller, which is responsible for the game logic of the mini-game and win/lose conditions.

The only interactable objects in the scenes are the bubbles, or the bubble behaviors, which have a color, speed and a lifetime. If the bubble is not popped before its lifetime has passed, the bubble will spontaneously pop, but no points are rewarded. Depending on difficulty level, more colors and bubbles will be present: the easy mode contains three colors and up to two colors can be popped, the medium mode contains six colors and up to three colors can be popped, while the hard mode contains all nine colors and up to four colors can be popped simultaneously. To avoid long waiting times before a bubble of the correct color appears, new bubbles are regularly spawned, their color depending on what colors are currently sparse.

#### 7.1.4. Breathing Mini-Game

Again, following the same structure as discussed in Section 7, the mini-game consists of a manager, responsible for the game objects and a controller, defining the game logic.

The only object in the scene is the space whale floating up and down. The objective is to breathe in and out at the correct time so the space whale is protected from falling comets. The water from the blowhole of the space whale responds to breathing. If the breathing follows the pace of the breathing instructions shown at the top of the screen, a reward will be given. The duration of the breathing mini-game is changed based on the selected difficulty level.

### 7.2. Architecture SpaceControl

The SpaceControl application, as shown in Figure 5, exists of two components, the User component responsible for retrieving all the available user and gaming data from the server, and a second component, the data graph component, responsible for displaying the retrieved data.

The SpaceControl app is created for Android devices, using the Android Studio IDE [58]. The MPAndroidChart Library [59], an Android library for chart views and graphs, is used to draw the different graphs incorporated in the SpaceControl app. To provide some basic security for the proof of concept, a basic authentication scheme is used for the HTTP connection between app and server.

The SpaceControl displays the data that has been collected during the gaming sessions of the child. The following lists the data that is logged while gaming:

- The mood at the start of the gaming sessions. This is a Likert scale, from 0 to 5 represented by smileys.
- A mood score from 0 to 5 collected at the end of a mini-game. Logged together with the type of mini-game that has just been played.
- The time and date of the gaming session.
- The duration of a gaming session.

### 7.3. Technological Choices

Two of the quality attributes the design of the system had to comply with, are performance and modifiability. To ensure that the waiting times remain low and transitions between different game states are minimized, the game is procedurally generated wherever possible. This way the same universe can be easily generated each time, containing the same mini-games on the same locations. Moreover, procedural generation allows developers to rapidly expand the game: it is not necessary to design different levels for each type of mini-game. Implementing a new type of mini-game is sufficient to provide a significant amount of new planets containing a unique version of the new type of mini-game.

More information about the technological choices made during the design and implementation process can be found in Appendix A.

## 8. Implementation

The following paragraphs will discuss implementation details of the relaxation module, with the senses and breathing mini-games in Section 8.1 and Section 8.2 respectively. Both these mini-games have been designed by consulting with specialized therapists. The following paragraphs will elaborate on how the relaxation techniques have been incorporated into the mini-games and which decisions were made based on the feedback of the experts.

For more information on the implementation of two non-relaxation mini-games and the SpaceControl application, Appendix B can be consulted.

### 8.1. Senses Mini-Game

The senses mini-game, visible on Figure 2a, focuses on the Visualizations or Guided Imagery technique, discussed in Section 2.1. Multi-Sensory Environments (MSE) were used as an inspiration for the design of the mini-game. MSE or “snoezelen” are defined as “*a room for relaxation where stimulation can be controlled, manipulated, reduced or intensified to fit the perceived motivation, interest, relaxation, therapeutic or educational needs of the user [60]*”. MSE is used by therapies for people with ASD and other developmental disabilities, dementia or brain injuries, to create a relaxing and stimulating environment [61]. Objects found in an MSE are designed to stimulate the user across a range of sensory modalities, e.g., fish tanks, bubble tubes, mirror balls and lava lamps.

The objective of the mini-game is to pop the bubbles of the correct color. Depending on the chosen difficulty level, more bubbles and more colors will be visible and the colors you need to pop will change at a faster pace. A larger score will be given for popping smaller bubbles. The total score will be displayed after the mini-game has ended. As the usage of only a smartphone limits the ability to incorporate sensory experiences, the focus is solely on auditory and visual stimulants. Therefore, it was decided to use lava lamps and the bubble popping sensation as inspiration for the mini-game.

Feedback from the therapists mentioned that the bubbles’ movement and noise should be the main focus of the game, to avoid loss of concentration or anxious feelings. Therefore it was decided to use a simple background, in the color purple, as this is often experienced as a calming and soothing colour [62], and a static foreground without any extra embellishing. The only moving elements in the scene are the colorful bubbles floating over the screen. It was important to find the correct balance for the speed of the bubbles to achieve a relaxing floating effect: movement should be fast enough to oblige the user to focus on the bubbles, but slow enough to avoid a nervous or anxious feeling when trying to tap the bubbles. In consultation with the therapist, specialized in relaxation techniques for children with ASD, a balanced pace has been established. Correct tapping of a bubble results in a popping sound, followed by a crackling noise for the collected space dust. Tapping an incorrect bubble is accompanied by a negative feedback sound and a tactile vibration. Throughout the mini-game, background music is played, but can be switched off separately from the sound effects in the settings.

To reduce fixation on the score, the total score is only displayed at the end of the mini-game. Only a zero-score or a positive score can be obtained. No points are deducted if an incorrect bubble is tapped.

### 8.2. Breathing Mini-Game

The breathing mini-game, visible on Figure 2b, incorporates focused breathing, which was discussed in Section 2.1. Different designs of applications for breathing training exist: a design based on only audio instructions, circle-based visualizations, displaying an expanding and contracting circle, wave-based visualizations, which use a wave-like line to show the optimal respiratory cycle over time, and, finally, visualizations of the human body, e.g., a 3D animated model of the human body expanding and contracting the chest and abdomen during breathing [63]. Providing visualizations can result in better engagement and can be perceived as a more relaxing experience [63].

The objective of the breathing mini-game is to inhale and exhale at the correct time. To decide on the appropriate breathing technique to use, a physiotherapist specialized in relaxation techniques, was consulted. She preferred diaphragmatic breathing over pursed lip breathing (PLB) as PLB requires the individual to pursue his/her lips together, tightening the lips, which she did not qualify as relaxation. To be able to detect when the user exhales, it was decided to let the user exhale while saying “ooom”. The water from the space whale’s blowhole will respond to the “ooom” sounds by going up, keeping Walter safe from falling comets. If the inhaling-exhaling sequence is performed correctly, the whale is rewarded with a snack, which results in points at the end of the game.

Another important aspect is the duration of the inhale and exhale. Children have a smaller lung capacity than adults, so too long periods would result in gasping for breath, while too short periods could lead to hyperventilation. Based on the experience of the therapist, specialized in relaxation for children with ASD, it was decided to opt for a 4-2-5 sequence: 4 s to inhale, a 2-s pause to hold your breath, followed by a 5-s exhale. The 2-s pause is needed to avoid hyperventilation or feeling rushed when practicing the breathing exercise.

The same background as for the senses mini-game is used and the foreground exists of only Walter the space whale, calmly swimming through space. At the top of the screen, the breathing instructions are displayed.

The only interaction in the mini-game, happens via the microphone. To detect the *ooom* sound during exhalation, the MicControl 3 [64] Unity asset is used. This asset uses the loudness value, i.e., the microphone input strength, to detect input noise. The microphone object is linked to the space whale, when noise is detected between a certain threshold, the water of the blowhole will go up according to the detected loudness. The calibration step at the start of the mini-game sets the thresholds according to the detected ooom. This is why it is advised to play the game in a calm environment, as noises from the environment can be detected.

When the mini-game is played for the first time, a calibration step will have to be completed first. You will need to inhale and exhale a few times as explained above, calibrating the smartphone’s microphone to your voice.

This mini-game contains no background music as microphone input is required. The only sounds presents are low rumbling whale sounds, played every few seconds.

## 9. Set-Up Usability Tests

To see if the proof of concept of the New Horizon game fit the needs of children with ASD and can be used as a tool for stress and anxiety detection, usability studies were conducted with three children and their parents. The usability tests took place over two weeks, during which the children and the parents had the opportunity to test the game and the app for the parents at home. Before and after these two weeks, a structured interview with one of the parents was conducted and questionnaires had to be filled in.

All subjects gave their informed consent for inclusion before they participated in the study. The study was conducted in accordance with the Declaration of Helsinki and has been approved by the Medical Ethics Committee of Ghent University on 16 March 2018 (Belgian registration number B670201835192).

### 9.1. Participants and Recruitment

**Figure 7:**
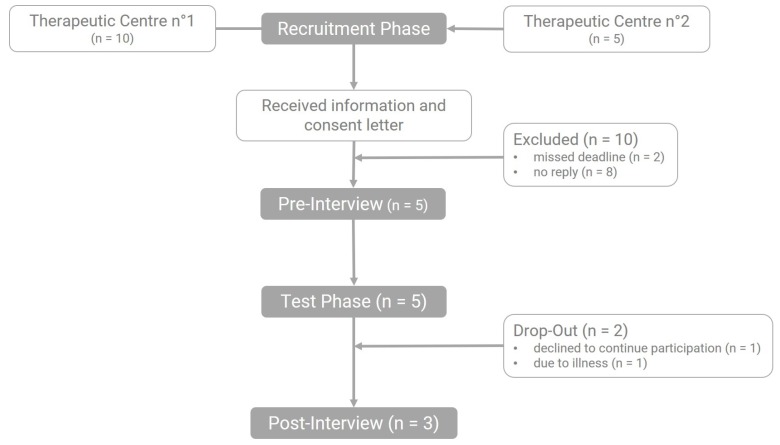
Participant flow diagram.

The recruitment took place via therapeutic centers. Twenty families, selected by the participating therapeutic centers, received an information and consent letter, five families agreed to participate. During the test phase two participants dropped-out, whereas three participants completed all phases. One of the participants dropped out because his mother believed the game was too easy for him, the other participant dropped out due to illness. Both dropped out before the test phase could be completed. Figure 7 gives an overview of the participant flow.

The participants were eligible to partake if they were between 6 to 10 years old, diagnosed with ASD, according to the DSM-V [3], did not have an intellectual disability and showed signs of stress and/or anxiety. It was preferred that the participants could read and liked to play video games. Participants were excluded from participating if parental permission was absent. Participants were provided with an Android device.

Some information about each participant has been collected during the interviews with the parents. An overview of each participant’s profile is given below:

- **P1** is an 8-year old female. Most difficulties are school-related, throwing a temper tantrum almost on a daily basis after returning home. Her mother indicates significantly fewer difficulties during holidays. She has no excessive need for structure, but social situations can be difficult. P1 has difficulties to express feelings of pain and is often too scared to mention problems at school. When feeling stressed or agitated, she often spontaneously retreats to her room or is provided with a distraction by her parents. She has mostly irrational fears, is unable to recognize dangerous situations and has trouble sleeping. She prefers YouTube over video games.
- **P2** is a 6-year-old male, unaware of his ASD. Most difficulties relate to a lack of structure, making holidays and weekends a challenge. The parents try to provide daily planning so P2 knows what to expect, to avoid temper tantrums. His mother indicates that they have yet to find an appropriate way to respond to these tantrums. Other characteristics are that he is often startled by loud noises, has difficulties to express his feelings and has often trouble sitting still. He sometimes expresses some negative thoughts about himself, but is overall considered an easy and happy child by his parents. His parents believe that a tablet or smartphone can be used for relaxation, but are afraid this would be a form of reward for unwanted behavior. He prefers watching videos of trains on YouTube over video games.
- **P3** is a 10 year old female. Her father reports difficulties regarding her emotionlessness and her anger, resulting in daily tantrums. The parents find it difficult to find an appropriate way to respond to these tantrums. Providing a calm and structured environment helps to keep her relaxed. Her father states that he believes she might often worry or feel scared, yet she hardly ever shares anything related to school or her feelings. Other characteristics are that she has trouble sitting still in case of lack of structure, difficulties with sleeping and negative thoughts towards her parents. She prefers reality series over video games.

### 9.2. Intervention

The experiment comprises two weeks of testing at home and a pre- and post-interview with a parent. Parents were asked to motivate their child to play on a daily basis and play when the first signs of stress or anxiety started to show. The parents were encouraged to use the SpaceControl app to keep an eye on the gaming behavior of their child. If the children started a gaming session after the encouragement of a parent, i.e., a non-spontaneous gaming session, parents were asked to write this down in the received User Guide.

The interviews took place at the therapeutic center without the presence of the therapists. Parents and children completed a questionnaire at home to bring to the interview. The pre-interview questions focused on the child and their difficulties or anxieties, whereas the post-interview targeted the gaming experience and possible improvements.

The questionnaires assessed the anxiety symptoms of the participants. The children, with the help of a parent, filled in the Dutch translation of the Spence’s Children Anxiety Scale (SCAS) [65]. The scale contains 45 items, which need to be rated on a four-point Likert scale, ranging from “never” to “always”. It comprises six subscales, e.g., social phobia and physical injury fears. It has good reliability and validity for the general population [66,67]. No validity has been established for the use for children with ASD, but literature shows that it is widely used as a measure for anxiety in children with ASD [11,68,69,70,71].

The parents filled in the Dutch translation of the Spence Children Anxiety Scale-Parents (SCAS-P) that measures anxiety symptoms of the child according to the parents [72]. The measure contains 39 items, the same items as the SCAS measure, but formulated from the perspective of the parents. It has good validity and reliability [73].

Information was collected about the gaming behavior of the participants during their gaming sessions. For each gaming session, the mood at the start of a session is collected. The user indicates mood on a five-point Likert scale, displaying smileys from very happy to very angry. For each mini-game, the mood, type, date and time is collected. This data will be sent daily to a server, for use in the SpaceControl data visualization app, discussed in Appendix B.1.

## 10. Results

The following sections will in detail analyze the game according to the different guidelines, list some performance values and discuss the results from the usability studies.

### 10.1. User and Parent Empowerment

In Section 3.1 some guidelines and best practices for the design of e-health applications for patient empowerment were listed. This paragraph will discuss these guidelines in reference to the design and implementation process of the New Horizon and SpaceControl applications.

- Design: the design process was an iterative, multidisciplinary, user-centered process, during which multiple specialized therapists have been consulted to provide feedback and information regarding the used relaxation techniques and needs of children with ASD. In total, three specialized therapists have been repeatedly consulted over a period of nine months.
- Implementation: the reason why a serious game has been used for this research, was because it provided the opportunity to reduce some of the limitations of CBT, namely a mobile serious game can be played anytime anywhere. The game can thus be incorporated into the daily life of the family and be used at home and during therapy, thus embedding it into the care process as a pervasive habit.
- Information content: over time, the SpaceControl app and its different graphs can provide valuable insight into the child’s gaming behavior and mood. However, for the parents to gain a deeper understanding of the child’s feelings, more different data and information should be collected, e.g., data from wearables to measure stress or anxiety or extra questionnaires throughout the day about the child’s mood or day.
- Awareness and acceptance: for the usability tests, the parents were informed about the study and its objective by the therapists and received a manual at the start of the experiment, to explain the game and its purpose in more detail. In a real-life situation, the application should contain all the necessary information to explain the used relaxation techniques and possibilities of the applications, so the user has permanent access to it.
- Relationship between patient and professional: at this moment the information exchange between parent and professional took place via the usual channels, e.g., weekly therapy sessions of the child or e-mail communication. However, the SpaceControl application could be extended to contain some form of communication, for example, weekly automated questions for the parents about the child’s behavior the past week. This way the therapist can focus on relevant topics during the weekly therapy sessions.

Overall, the first steps towards empowering both the child and the parent have been taken with this first prototype of the New Horizon and SpaceControl application. Nonetheless, there are plenty of improvements possible to offer a system that is usable and effective in the daily lives of families coping with the day-to-day challenges of ASD.

### 10.2. Game Analysis

In Section 3.2 the game attributes relevant to learning were discussed and in Section 3.4 and Section 5 the guidelines for designing a serious game for children with ASD were discussed. This section will provide an analysis of which of these elements are present in the New Horizon game and how the elements for learning and guidelines for ASD can be contradictory.

Table 6 provides an overview of which of these game elements are present in the New Horizon game, together with a short explanation or example for each attribute. In total, 12 out of 18 attributes can be found in the New Horizon game in some shape or form. Table 7 indicates which of the guidelines found in the literature have been applied to the New Horizon game.

Comparing the game elements that contribute to learning with the guidelines found in literature and assembled by the therapists, indicates that a more modified approach is needed when designing a serious game for children with ASD than simply incorporating the elements for learning. Some of the guidelines for ASD discourage the use of certain game elements whereas others reinforce it.

Game elements that are important to enhance the immersion of the player are the environment, game fiction and immersion, more specifically the game attribute sensory stimuli which may enhance this feeling of immersion in an alternate reality of the game. These game elements are elements related to the background story of the game. A background story is often an important element of a game, as it helps make sense of the objectives of the game and the world in which the game is set. However, it can also be an important element to keep the player interested in the game as he/she discovers more about the characters in the game or the world as he/she progresses throughout the game. However, for children with ASD, a background story can become an unwanted distraction. Too much information about the characters can lead to unwanted obsessions leading to the diversion of the actual goal of the game. For children with ASD, the background story of the game should be reduced to a minimum and only used to make sense of goals and challenges in the game. Sensory stimuli should be reduced to functional minimal graphics and audio.

Another important game element, that is a major contributor to the intrinsic motivation in digital games is conflict and challenge, which is the presentation of the challenges in a game and the nature and difficulties of these challenges. More specifically, the game attribute surprise contains the degree of uncertainty in these challenges. For children with ASD, the game must be repeatable and predictable. The player should be able to enhance its skill level in the game, by coming back to certain parts of the game and replaying them multiple times. The gameplay should become predictable to the user after playing for a while and should contain no unambiguous actions that lead to unexpected game behavior. The challenge herein lies that, even though these elements should be kept functional and predictable, a certain level of wonder or surprise still has to be present in the game, creating a balance between predictability and surprise, resulting in serendipity to assure the player will remain interested in playing the game.

A final game element that has very little use in a serious game for this purpose is human interaction. In Section 2.2 the modifications of CBT for ASD were discussed. One of its limitations was that therapy sessions focus on verbal face-to-face communications, which is often an obstacle for people with ASD. The game can be used in therapy, requiring some face-to-face interaction, however, no human interaction is necessary to play the game.

The guidelines from the therapists and literature have also shown that certain game elements become more important when designing for children with ASD. The game element assessment provides the player with feedback regarding his progress in the game or guides the player towards certain game goals. For children with ASD, scoring and feedback should be used as a tool to motivate the player to continue playing, even though his/her performance was mediocre. Important to note is that, although assessment and scoring is an important game element for children with ASD, this is only effective when it is limited to positive feedback and scoring. Incorporating negative feedback or scoring can have opposite results, e.g., loss of interest or increased stress and anxiety.

A final important game element to incorporate is rules and goals. It is self-explanatory that every type of player should be aware of how to make progress in the game. However, for children with ASD, each gaming session must be limited to one goal, which should be reinforced throughout the gaming session. An important caveat here is that the instructions to clarify this goal should be stated explicitly for children with ASD and free from any figure of speech or text that can be misinterpreted. If this is not the case, the instructions could lead to confusion, frustration or temper tantrums. To avoid incorporating too complex instructions, a specialized therapist, used to working with children with ASD of the targeted age category, should have final say of any text incorporated into the game.

### 10.3. Performance Tests

In Section 6 the most important quality attributes of the system were discussed. As it is important for children with ASD to avoid unwanted obsessions as a result of prolonged static images or loading animations, transition times between different game states have to be minimized. To counteract this, both the game and the mini-games are procedurally generated.

**Figure 8:**
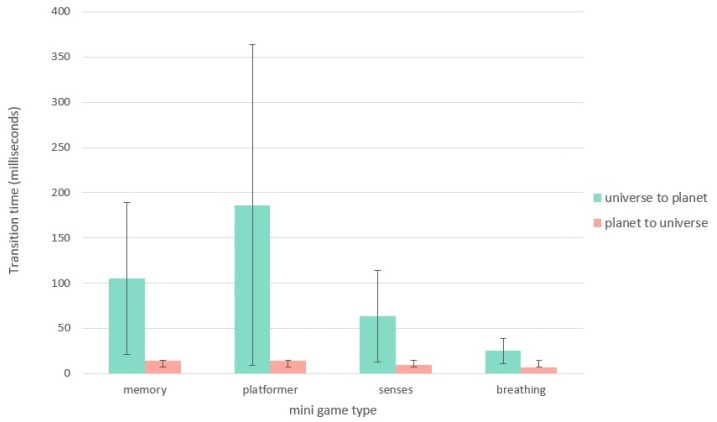
The average of five measures, in milliseconds, for the transition times between universe and planet for all mini-game types. The tests were performed on a Google Pixel XL device with Android 9.

During a game session, no loading screens are visible when going from universe to planet and back. Figure 8 gives an overview of the loading times for each of the mini-game types. For each type, the transition time, in milliseconds, is displayed for both transition from the universe to the planet and back. All of the transitions remain below 0.2 s, resulting in an almost instant transition between gaming states. The platformer mini-game shows a significantly higher transition time for landing on a planet. This can be attributed to the more complex gaming structure of the platformer challenge, containing not just one scene, but a selection of aggregated zone elements that need to be placed in the correct order before landing. The high standard deviation could also be attributed to the zone generation, as some levels will contain more diverse zone elements while others might contain more identical building blocks.

**Figure 9:**
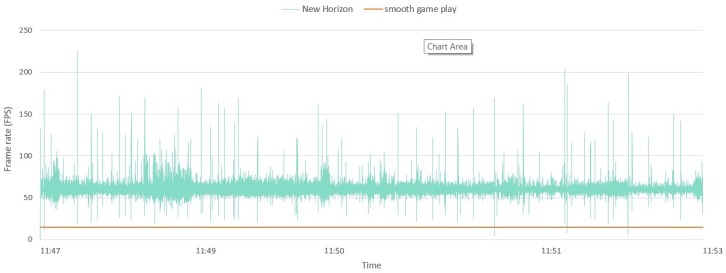
The frame rate in fps during a gaming session. During the gaming session, the player has flown his spaceship through the universe and has played each type of mini-game once. To achieve ‘smooth’ gameplay, the frame rate has to be higher than 15 fps. Test performed on a Google Pixel XL with Android 9.

Figure 9 displays the frame rate throughout a gaming session. During the gaming session, the player flies through the universe with his spaceship and plays every type of mini-game. As visible on the figure, the frame rate averages around 60 fps, with some drops below 15 fps. To maintain a smooth gaming experience, the recommended frame rate is 15 fps or higher [74]. The performance will thus remain high throughout the gaming session.

### 10.4. Usability Tests

In Section 9 the set-up of the usability tests was discussed. This section will present the results of those tests. First, the post-interviews will be discussed, followed by the results of the SCAS questionnaires. Finally, the collected gaming data will be analyzed in detail.

#### 10.4.1. Post-Interview

After two weeks of testing at home, the parents were asked to return for another interview to discuss how they experienced both applications. The SCAS and SCAS-P questionnaires were again filled in before coming to the interview.

A first problem that became clear from all the interviews was that the parents regularly or completely forgot to track the moments of non-spontaneous play. During the first interview, they were asked to write down the time and date when their child played the New Horizon game after parental encouragement. Combined with the game data this could indicate if the game was played spontaneously or not. However, none of the parents remembered to do so.

Second, two of the three parents handed the smartphone to their child, while the third parent remained in control of the smartphone and handed it over when the child asked to play the New Horizon game, which was on a daily basis. The other two children might have been more interested in the smartphone itself than the New Horizon game specifically, although they played the game after the encouragement of the parents and sometimes spontaneously. As a result of handing the smartphone to the children, the parents did not use the SpaceControl app at all. The third parent had WiFi issues, therefore the data was unable to synchronize. Unfortunately, she did not respond to repeated inquiries from the therapists about Wi-Fi connections, thus the data was only synchronized after the experiment had ended.

Next, the parents often encouraged the children to play the game, however, only when they were calm and relaxed. They felt it would be inappropriate to let them play a game when they were throwing a temper tantrum.

Finally, the breathing game was often experienced as boring and by one participant as too difficult and the parent suggested some more practice might have been needed. The platformer, on the other hand, was too difficult to complete. One participant even refused to continue playing, after playing daily for a week, because of being stuck in the platformer challenge.

#### 10.4.2. Spence Children’s Anxiety Scale

Some issues arose with the SCAS questionnaires. One parent forgot to fill in the questionnaire before the pre-interview and was asked to fill it in as soon as possible and bring it to the post-interview, together with the post-interview questionnaire. However, this resulted in identically filled in questionnaires. The other participants returned incorrectly filled in lists, as some questions were left blank, where others contained multiple circled answers. These questions were left out of the calculation of the total score. One of the children even wrote on the questionnaire that *“she did not want to say anything anymore”*, resulting in always circling “never” for the remaining questions.

The results of the SCAS and SCAS-P of the pre- and post-test can be found in Table 8. A T-score of 60 or higher, indicates elevated levels of anxiety. The results show that according to the SCAS questionnaire for children, only one participant shows elevated levels of anxiety during the pre-test. If the T-scores of the subscales are examined, each child shows elevated levels of anxiety for at least one disorder. The T-scores of all participants, except P2 due to identically filled in questionnaires, show a decrease in T-score after the experiment. The results of the parent version show similar results before and after the tests.

Since the P1 and P3 have only played the game sporadically, the decrease in anxiety is more likely to be attributed to the fact that the SCAS questionnaire might not be the ideal measure for anxiety detection in children with ASD, as parents reported it was challenging to complete the questionnaire together with their child. Other questionnaires exist that might be more suited for young children. The Revised Children’s Manifest Anxiety Scale (RCMAS) [75] is a self-report measure for chronic anxiety and consists of 37 yes or no questions. The Children’s Automatic Thoughts Scale (CATS) [76] is a 40-item scale that assesses a range of negative self-statements in children and adolescents. A combination of these measures could prove to be more useful for anxiety detection in children with ASD.

#### 10.4.3. Collected Data

**Figure 10:**
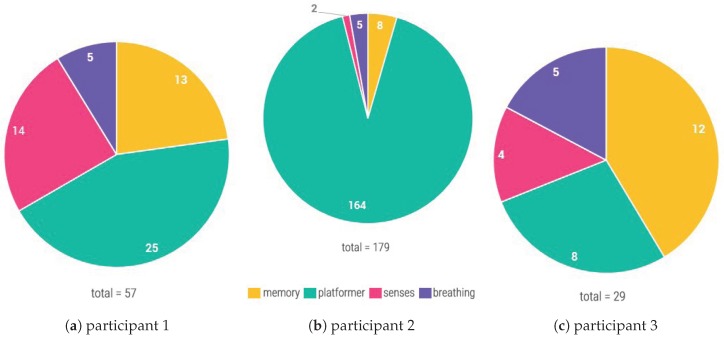
The mini-game rates.

The collected data has been analyzed and displayed into three graphs for each participant. The first graph, shown in Figure 10, shows the pie chart of the number of times each type of mini-game has been played by each participant. For two participants, shown in Figure 10a,b, the platformer is the most played type of mini-game, with P2 having played the platformer 164 times. The memory mini-game is the second most popular game for most of the participants, except for P3, shown in Figure 10c, while the breathing mini-game is the least played type of mini-game.

Important to note is that none of the participants managed to complete the platformer mini-game. The large amount for P2 could indicate that this mini-game provoked repetitive behavior in P2.

One of the parents indicated that the platformer on the easiest difficulty level was still too difficult, whereas the senses mini-game became too easy. Switching between difficulty levels during gameplay was a nuisance. This could indicate why the senses mini-game has not been played more often.

**Figure 11:**
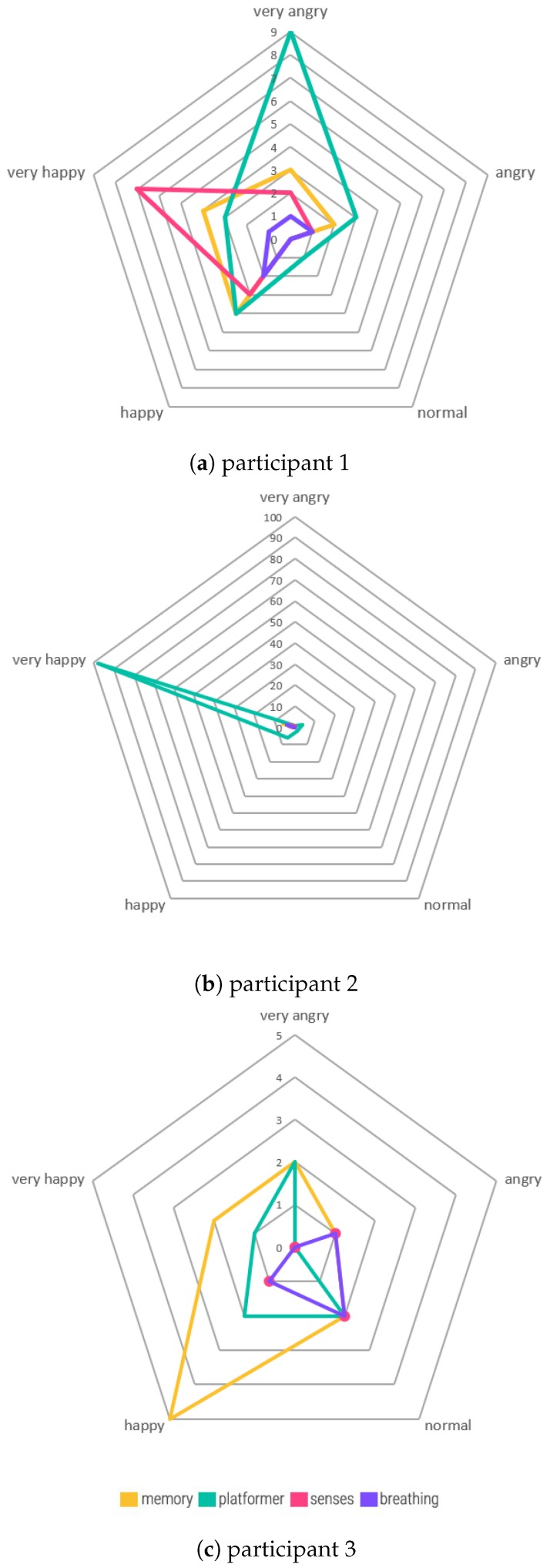
Relationship between mood and mini-games.

In Figure 11, the link between the mood and the type of mini-game is visible.

The platformer mini-game, that none of the participants managed to win, resulted nonetheless in a limited angry mood. The results of P1, shown in Figure 11a, show frustration after playing this mini-game, but P2 and P3, shown in Figure 11b,c respectively, remain overall positive. P2 even continuously indicates a very happy mood, which is in agreement with his mother, who portrayed him as a very happy child that enjoyed to play the New Horizon game, however, it is important to keep in mind that he could have chosen the same smiley over and over again for continuity.

Overall, the results of the radar chart show that none of the mini-games consistently leads to frustration, except for P1, which can be explained by repeatedly losing.

**Figure 12:**
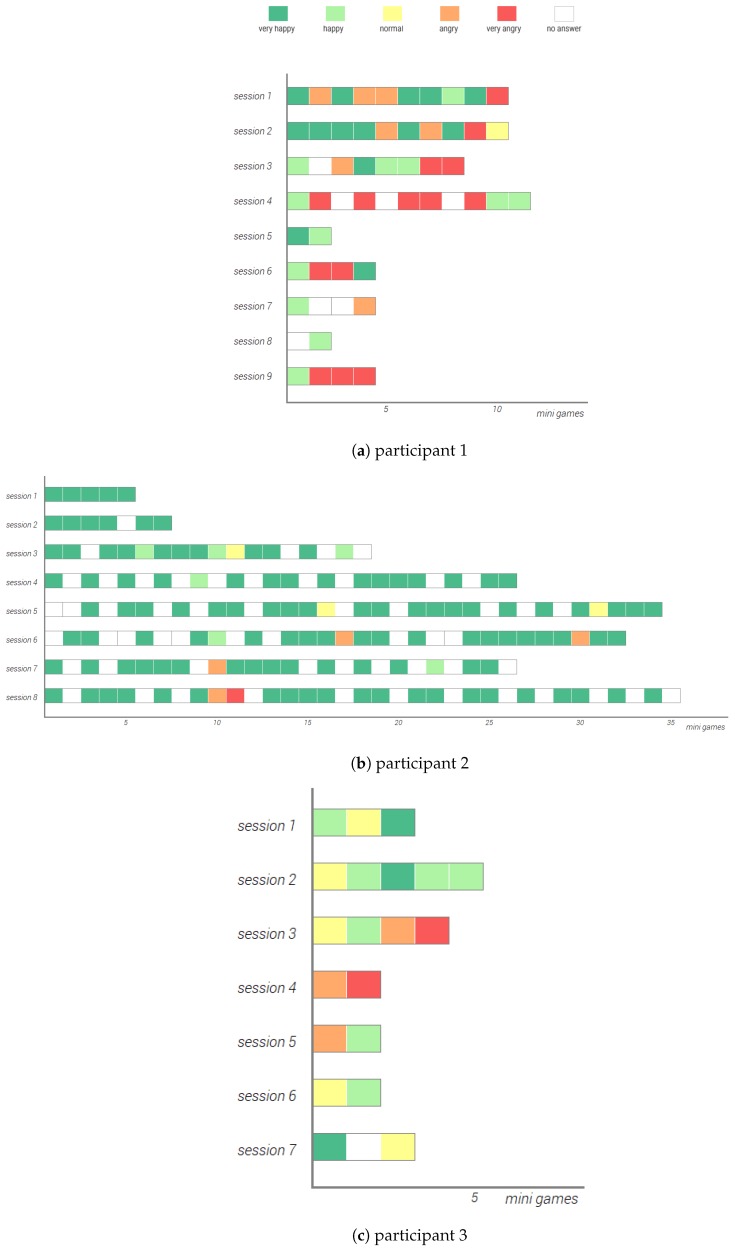
Evolution of the mood during a gaming session.

The color chart in Figure 12 show the indicated mood for each mini-game during a gaming session. The vertical axis indicates the sessions, the horizontal axis shows the total number of mini-games in each session and the color of a cell indicates the mood.

Due to problems with saving the mood, the mood at the start of a gaming session was missing for most of the gaming session, therefore, this mood has been omitted for the chart. For some of the challenges, there was no mood recorded after finished a challenge, it is believed this is due to participants turning the game on and off to avoid indicating their mood.

The chart shows no real increase or decrease in the mood over the evolution of a session. This can be attributed to the fact that none of the participants played the game when feeling stressed or anxious. As can be seen in the chart, most sessions were started with the participants indicating an overall happy mood. P1, shown in Figure 12a, show more negative feelings compared to P2 and P3, shown in Figure 12b,c. These negative feelings can be linked to the difficulty of the platformer mini-game, as seen in the previous graph.

The chart of P2 shows longer gaming sessions compared to others, combined with the knowledge that he almost only played the platformer, this can again indicate repetitive behavior.

## 11. Discussion

Numerous studies incorporate some form of technology to address the problems and difficulties related to ASD for children, of which most focus on language skills and emotion recognition. There are only a few studies that discuss the use of a serious game for stress and anxiety reduction in children with ASD, however, no results have yet been reported and none involves the parents. This study investigates if a serious game can be used for stress and anxiety reduction and if a parental application can give insight into the child’s feelings.

It is important to note that the findings presented remain limited. Both applications have only been tested for two weeks by three families. Since children with ASD and their systems are unique, it is important to test the applications on a larger scale. However, some modifications can already be made based on the results of these three families, which will be discussed below.

The results of the usability studies show, that although distracted by owning their own smartphone, the children enjoyed playing the New Horizon game, the memory game was the most popular mini-game whereas the breathing mini-game was mostly deemed too boring or difficult to play spontaneously. This can be avoided by incorporating the breathing mini-game as an obligatory exercise in cases of stress. Including the game in a therapy session can increase opportunities to practice breathing skills and educate the parents about the benefits and therapeutic use of the game.

Overall, some positive effects of taking the guidelines for patient empowerment into account are visible. First, the multidisciplinary approach and the guidelines for ASD have resulted in a game that fits the needs of children with ASD and limited unwanted behavior has been noted. Although never playing the game when feeling stressed or anxious, the results of the Likert scale also showed the children were generally in a happy mood, indicating limited frustration as a result of the game. Only one participant refused to continue playing as one of the mini-games was too difficult to complete, which can be easily fixed by adding a hint-based system to this mini-game or adding the option to exclude a certain type of mini-games from the universe if it triggers unwanted behavior. Second, some participants have already shown some form of habit-forming as they often played the game returning from school. Nonetheless, to create actual awareness and acceptance for the parents, the information content of the SpaceControl needs to be extended, reminding them daily to check on their child’s mood and gaming behavior. Finally, it also became clear from the usability studies, that more attention needs to be paid to engaging the parents in the process and reminding them on a daily basis to use the SpaceControl application. This was not the case at the moment, since two out of three parents handed the smartphone to the children.

The results of this research provide some answers to the three investigated hypotheses, however, future modifications will be necessary to fulfill the hypotheses.

First, the children did indicate reduced levels of stress and anxiety after the study, however, the post-interview showed that none of the children played the game when feeling stressed or anxious. Moreover, the SCAS anxiety scale might not be the ideal measure for stress and anxiety detection in children this young with ASD. At the moment, no wearables were included since the usability of the current set-up needed to be evaluated first. However, incorporating non-intrusive wearables, such as a smartwatch, could be an added value for both applications, as it could provide a more objective and accurate analysis of the anxiety and stress of the child. Taj-Eldin et al. [77] and Torrado et al. [78] give an overview of existing wearables and feasibility for physiological and emotional monitoring for people with ASD. Incorporating the physiological data in the SpaceControl app could provide the parents and therapists with a more understandable and objective overview of the child’s anxiety and stress in relation to the game data.

Moreover, the wearables can be incorporated in the game itself. When a wearable detects the child is anxious or stressed, the breathing exercise should be completed first before other types of mini-games can be played. Ensuring modifiability, the universe and mini-game components are completely separated, making it easy to add an option that analyses the physiological signals of the wearable, before the universe, and the planets in the close surroundings, are generated. No changes to the existing mini-games need to be made.

Second, it was tested if the child adopts the game as a tool for stress and anxiety and continues to use it spontaneously. Although one of the children was asking to play the game on a daily basis, none of the children had the chance to play the game when feeling stressed or anxious. This can be attributed to the hesitance of the parents to encourage their children to play a game when throwing a temper tantrum. By incorporating the game in the therapy sessions, not only will the children learn to use it when they are feeling stressed and anxious, the parents will learn to see the game more as a tool to reduce stress and anxiety. Moreover, during this entire process, the expertise of the therapists was consulted, to create a game that fulfills the need of children with ASD. However, the results show that this does not necessarily lead to a fully immersive and engaging system. To create a game that can both create relaxation and enjoyment in children with ASD, it might be necessary to not only include experts, but also children with ASD in the design process. Malinverni et al. [79] proposed a model for such an inclusive design approach for developing therapeutic games for children with ASD. The model sees the structural elements of the game, defined by the experts, like empty boxes that are filled with specific elements using the contributions of the children. For example, in the breathing mini-game, the structure provided by experts is the used breathing technique, diaphragmatic breathing, while the children come up with the space whale.

Finally, no conclusions can be made in regards to the supporting SpaceControl application as none of the parents has properly tested the application. To create an experience that empowers the parents to manage their children’s temper tantrums and anxiety, parents should also be included in the design process of the SpaceControl application. Via interviews and mock-ups the desires and expectations of the parents can be modeled into specific application features.

## 12. Conclusions

This research aimed to design a prototype of a serious game for children with ASD to reduce stress and anxiety and to evaluate the feasibility of designing technology for the empowerment of children with ASD and their parents. The results show that, although some guidelines are already available in the literature for patient empowerment, more research needs to be done on how to practically incorporate these into e-health applications. The process of designing an application for the empowerment of families with children with ASD is an iterative process that requires the input of all parties involved. Moreover, this research shows the importance of regular user testing and a multidisciplinary approach, when designing for a target group with specific requirements to create an application that represents the needs of the target audience.

## Figures and Tables

**Table 1:** Guidelines regarding the modification of Cognitive Behavioural Therapy (CBT) for Autism Spectrum Disorder (ASD) according to National Institute for Health and Care Excellence (NICE) [34] and literature reviews [11,35].

Recommended Modifications of CBT for the Treatment of Anxiety Disorders in Children with ASD
1. Disorder specific hierarchies: CBT should not solely focus on the problem it needs to treat, i.e., anxiety, but it should also incorporate disorder-specific problems of ASD, such as training of communication and social skills and emotion recognition, which often lack in children with ASD.
2. Concrete, visual tactics: abstract language and concept usage should be reduced by incorporating visual material to accommodate the children’s literal mindset, e.g., drawings and pictures, narratives and social stories. Devoting a larger part of the sessions of exposure and relaxation techniques places less focus on the need for sufficient communication skills.
3. Child-specific interests: children with ASD often have specific interests, which can be unusual and excessive. Using these interests in CT can increase their motivation and enjoyment of the therapy sessions. It is, however, important to find a balance as not to reinforce possible problematic obsessions when using these specific interests during therapy.
4. Parent involvement: if parents are involved in the CBT treatment, the generalization of learned skills increases via practice at home. Parental involvement in the sessions also decreases the possibility of accommodating the child’s behavior at home as parents learn to apply these techniques at home.
5. Repeated content: match the duration of the session to the child’s pace and repeat content to aid learning.
6. Rating scales: children with ASD are often unable to express their emotions. Use rating scales or a feelings thermometer to measure a change in their mood instead of verbally asking how they are feeling.
**7. Play**: Games can be used during therapy sessions to explain and learn concepts and keep the interest and motivation of the children

**Table 2:** Alpay et al. summarizes guidelines for developing e-health applications for patient empowerment [12].

Guidelines and Best Practices for the Development of Patient Empowerment E-Health Applications.
1. Design: using a multidisciplinary and user-centered design approach can ensure that both the needs of the patient and the healthcare professionals are being met.
2. Implementation: embedding e-health in the care process and reducing identified barriers that prevent these applications from getting past the pilot phase.
3. Information content: collecting quality information for users and patients is only the first step and takes time. The focus should be on information usability during the development process.
4. Awareness and acceptance: the patients should be aware of the possibilities and added benefits of using e-health applications.
5. Patient and professional relationship: channels for e-communication and information exchanges should be in place.

**Table 3:** Game elements and their respective attributes relevant to learning [15,36,37,38].

Overview of the Game Elements and Attributes in Serious Games That Can Lead to Learning
Action language comprises the communication rules of the game between player and system. The attribute language/communication of the game is the method by which the player can make their intent clear to the system, e.g., use of arrows on the keyboard.
Assessment refers to the nature and content of any feedback during the game. This feedback provides the user with information about his advancement towards certain game goals. Feedback is the most effective when it does not interrupt gameplay. Assessment is the measurement of achievement used in the game, e.g., scoring, while progress is the advancement of the player towards certain game goals.
Conflict and challenge refer to the presentation of problems and challenges and the nature and difficulty of those problems. This category is a major contributor to the intrinsic motivation inherent to digital games. Challenge refers to the difficulty of the game, conflict is the nature of challenges presented to the user, e.g., violent versus non-violent, verbal versus kinetic. Surprise is the amount of uncertainty in these problems. Finally, adaptation is the degree to which the game’s difficulty can be altered based on the player’s actions. Ideally, this should be adapted according to the player’s accomplishments and skill level to ensure optimal player engagement.
Control is the degree of actual interaction and activity of the player. The capability to control pace or level of challenge throughout the game and receive immediate feedback helps to sustain the engagement of the player and potentially enhance the likelihood of learning. The attribute control refers to how much the player can regulate or manipulate certain aspects of the gameplay, e.g., manipulate in-game objects like doors. Interaction (equipment, on the other hand, is the amount of change that takes place in the game as a result of the player’s actions.
Environment and the attribute location entail the physical world in which the game is set. This provides context for the game, which can enhance the immersion of the player in the game. It is closely linked to game fiction.
Game fiction describes the nature and story of the game world. Game fiction describes how the game world is presented to the user: Is it realistic or fantastic? Does the player know this? Are his/her actions and consequences represented directly or indirectly? The game world is a tool for the player to make sense of the world. Fantasy are the elements that are dissimilar from the real world, while mystery refers to the gap between known and unknown information.
Human interaction refers to any human-to-human contact in the game. Interaction (interpersonal are the relationships between players in real-time and space, e.g., face-to-face contact and interaction (social) refers to interaction via technology, e.g., instant messenger communication.
Immersion describes the player’s perception and affection towards the game fiction. It differs from game fiction as it describes how the user perceives the game world and his/her place therein, whereas game fiction describes the game world. Immersion is the degree to which the player feels he is an agent within the game world. It occurs when the player experiences a balance between the game challenges, his/her ability to play the game and current knowledge about the game. Representation describes the perception of the player’s self in the game’s reality, while sensory stimuli refer to the auditory and visual stimuli, which may enhance the acceptance of an alternate reality during the course of the game. Pieces and players are the game objects or people included in the game narrative. Safety provides the disassociation of actions and consequences: actions within the game have no real-life consequences, which allows the player to focus on the game itself.
Rules and Goals describe the degree to which the game has clearly defined rules and goals and how to make progress towards these goals. It contains the reason for which the player interacts with the game world and the motivation for his/her in-game actions. This category also contains the rules on how the user can solve these problems or reach these goals.

**Table 4:** An overview of the guidelines found in the literature regarding the design and implementation of software or serious games for children with ASD [22,49,56,57].

Guidelines from Literature for the Design of Software for Children with ASD.
1. Customizability: every child’s ASD symptoms manifest uniquely. What might work for one child, might not work for another. The gaming experience must be highly adaptable to the child’s individual preferences and skills: the children, parents or therapists should be allowed to customize feedback and rewards, avatars, the combination of text, audio or video options, the visual elements of the game and the game content.
2. Evolving tasks: increasing levels of motor or cognitive complexity should be incorporated in the game. Game sessions should involve similar activities, but when the necessary skills are acquired, they grow more demanding to enable the progression of the learned skills.
3. Unique goal: there should be one unique explicit goal to reach within a gaming session.
4. Instructions: goals and tasks should be clear before playing and should be reinforced during the whole gaming session. Instructions and commands should not rely heavily on text or language.
5. Reward: offering a reward after a good performance, increases the child’s motivation, engagement and implicitly improves skills. Children with ASD prefer rewards that create fun, e.g., cheerful music and animations over quantitative rewards, e.g., points or extra time. Penalties should be omitted when the gaming performance is mediocre and replaced by messages that motivate the children to do better next time.
6. Repeatability and predictability: repeatability is important to master a skill and to provide control of the learning rate. Repeating the same tasks creates certain predictability of the expected game goals for the next gaming session.
7. Transitions: transition time between different game states should be minimized. If transition times become long and complicated, the child might lose his/her attention during the transition. Transition screens should be kept simple, no sound or animations, to avoid fixations on repetitive elements. Some children can become fixated on repetitive elements, resulting in constantly turning the game on and off instead of playing.
8. Minimalistic graphics: graphics should be aesthetically pleasing, but always functional. Irrelevant elements might form a distraction and can lead to loss of attention. Too many visual or colors might trigger anxiety as it might be difficult for the child to interpret individual elements.
9. Clear audio: children with ASD can be sensitive to audio stimuli, which can create extra stress. Sound or music can be used to provide feedback on actions, to complement a visual reward or during a transition phase in the game. The same principles as for visual elements apply and audio should always remain functional. Nursery rhymes or classical music are preferred if music is used throughout the game, although music should always be optional.
10. Dynamic stimuli: providing animations or music helps to retain the child’s attention. If there are no visual or auditive stimuli, the child might lose his/her attention. A prolonged static visual, on the other hand, might trigger unwanted behavior, such as stereotyped movements or motor rigidity, e.g., gazing at a static image on the screen.
11. Serendipity: visual or audio effects can create wonder or surprise, resulting in increased enjoyment of the game. This is also true, to some degree, for children with ASD: sensory stimuli should be predictable and consistent with certain tasks, e.g., audio feedback for a correct action with some serendipitous effects, e.g., a new object that appears on the screen. It is important to find a balance between sensory stimuli to avoid attention loss or unwanted behaviour.

**Table 5:** Guidelines for the design of serious games for children with ASD, based on the remarks and feedback during multiple feedback sessions with specialized therapists.

Guidelines for the Design of Serious Games for Children With ASD Based on the Expertise of Consulted Specialized Therapists.
1. Sound and music: it should be possible to turn off the music and the sound effects separately and at all times. Turning off the sound or music should not interfere with gameplay, e.g., if negative action is accompanied by a sound effect, it should still be clear that it is a negative action when the sound is turned off, by, for example, adding a vibrating effect.
2. Background Story: the presence of a background story does not interfere with the gameplay if it is used to explain game elements or goals, but should remain limited. For example, mentioning that Jimmy is exploring the universe and lands on planets to relax or regain some energy, helps explain the course of the game. If the background story becomes too extensive, it can lead to loss of concentration or the children might become more focused on the story than the actual game. For example, adding a complex origin story for Jimmy can distract from the objective, which is to play the mini-games when feeling stressed.
3. Language and text: any language and text present in the game should be free from figures of speech and as clear as possible, e.g., “watch out, you can’t swim!” can be confusing as the child might believe “you” refers to him/her and not Jimmy in the game, which can lead to a confused reaction such as “but I can swim!”.
4. Actions and goals: the goals of each mini-game should be clearly defined and the actions that need to be taken to reach those goals should be unambiguous, e.g., if you hit the correct star in a memory mini-game, this should be reinforced via visual and sound effects and this should happen every time a correct star has been hit.
5. Simplicity: the mini-games designed for stress and anxiety reduction should contain visual simplicity. Only a limited number of colors should be used and the game settings should be limited to solely functional game-objects and sounds. If the game is too visually complex, the children might receive too many sensory impulses, which can lead to loss of attention or increased anxiety and stress.
6. Scoring: scoring should never be negative and all negative text should be minimized. For example, points for completing a mini-game should not be taken away by assigning negative points for every failed mini-game. If the player is unable to complete a mini-game, one should still feel motivated to continue playing the game, e.g., instead of “you lost!” a more positive wording should be chosen, such as “you did your best, better luck next time!”.

**Table 6:** Analysis of the game elements integrated in New Horizon.

Category	Definition	Attributes	New Horizon	Explanation
Action Language	The method and interface that allows the player to interact with the system.	Language/Communication	**✓**	Touch input
Assessment	The nature and content of any feedback given to the user during the game	Assessment	**✓**	Points and spaceships
		Progress	**✓**	Pop-up visualizing what spaceships have already been rewarded and what not
Conflict/Challenge	The presentation of problems and challenges in a game and the nature and difficulty of those problems.	Adaptation	**✗**	No in-game difficulty adaption based on player actions
		Challenge	**✓**	Difficulty level can be set via the settings
		Conflict	**✓**	Mini-games are non-violent and kinetic, accessed by landing on a planet
		Surprise	**✗**	Only four types of mini-games exist, each with a fixed objective. The user will know what to expect of each mini-game after a while
Control	The degree of actual interaction and activity of the player.	Control	**✓**	Limited objects are interactable, e.g., bubbles, stars
		Interaction (equipment)	**✗**	Interactable objects are linked to scoring and rewards, no change in game-play
Environment	The physical world in which the game is set.	Location	**✓**	Outer space
Game Fiction	Describes the nature and story of the game world.	Fantasy	**✓**	The game world is fantastic
		Mystery	**✗**	The player knows the world is fantastic and his actions are represented directly. He/she knows what actions to take to complete a mini-game or earn a new spaceship
Human Interaction	Describes the human-to-human contact within the game.	Interaction (interpersonal)	**✗**	It is a single-player game
		Interaction (social)	**✗**	It is a single-player game
Immersion	Describes how the player perceives the game world and himself in the game.	Pieces or players	**✓**	Limited to Jimmy (player) and his spaceship and space whale Walter
		Representation	**?**	Subjective. The player is the character Jimmy in the game
		Sensory stimuli	**✓**	The game contains audio effects throughout the game that support the visual world, e.g., spaceship motor sounds
		Safety	**✓**	In-game actions have no real-life consequences
Rules/Goals	Describes how the player can achieve the goals of the game.	Rules/Goals	**✓**	The goal and how to reach it is shown at the start of each mini-game. Extra info can be found in the user manual via the settings.

**Table 7:** Analysis of the design guidelines integrated in New Horizon.

Category	New Horizon	Remarks
Customizability	**✗**/**✓**	No customizability of game content. Select difficulty level, controls of spaceship and audio preferences in settings. Player can choose to ignore the least favorite mini-games.
Evolving Tasks	**✗**	No automatic adaptation to skill level, limited difficulty levels, selected via settings menu
Unique goal	**✓**	Each mini-game has a unique goal. The global goal of the game is to collect all spaceships by completing the mini-game goals.
Instructions	**✓**	The goal and task of each mini-game are explained at the start of every mini-game. No audio instructions, only text.
Reward	**✓**	Points rewarded for completing mini-games. A new spaceship is awarded for breaking a high score or winning 3 consecutive mini-games. In a mini-game, rewarding audio and visual effects are present. No reward is given if the mini-game is lost.
Repeatability and predictability	**✓**	Mini games can be repeated as often as the player wants. The goals of the specific type of mini-games remain the same.
Transitions	**✓**	No loading screens between universe to mini-game transitions.
Minimalistic graphics and clear audio	**✓**	The graphics and audio for the mini-games in the relaxation module are functional. Audio and music are optional throughout the game.
Dynamic Stimuli	**✓**	All mini-games contain dynamic stimuli. The only static scene in the game is player-induced, when the user is not moving his spaceship in the universe and there are no satellites in the vicinity. This is however not a situation that the player needs to undergo in specific situations.
Serendipity	**✗**	Sensory stimuli are predictable and consistent with a certain task. Little to no wonder or surprise if the user has been playing the game for a while, satellites can be seen as an element of surprise.

**Table 8:** T-scores of the Spence’s Children Anxiety Scale (SCAS) and Spence Children Anxiety Scale-Parents (SCAS-P) questionnaires.

	SCAS						SCAS-P					
	Pre-test			Post-test			Pre-test			Post-test		
	P1	P2	P3	P1	P2	P3	P1	P2	P3	P1	P2	P3
panic attack + agoraphobia	55 *	≤40	55 *	55	≤40	≤40	65 ^•^	52	63 ^•^	65 ^•^	52	65 ^•^
separation anxiety	68 ^•^	≤40	65 ^•^	60 ^•^	≤40	49	58	44	63 ^•^	56	44	49
physical injury fears	63 ^•,^*	64 ^•^	65 ^•^	50	64 ^•^	60 ^•^	51	60 ^•^	≥70 ^•^	51	60 ^•^	61 ^•^
social phobia	57	≤40	≤40 *	60 ^•^	≤40	≤40	63 ^•^	45	48	63 ^•^	45	48
obsessive compulsive disorder	45 *	≤40	50	≤40	≤40	45	64 ^•^	50	≥70 ^•^	58	50	≥70 ^•^
generalized anxiety	65 ^•^	≤40	≤40	45	≤40	44	52	≤40	60 ^•^	52	≤40	52
**total**	60 ^•^	≤40	52	52	≤40	42	60 ^•^	42	64 ^•^	58	42	63 ^•^
**percentile (%)**	84	≤16	59	55	≤16	19	84	22	91	80	22	90

^•^ A T-score of 60, or higher, indicates elevated levels of anxiety. * Incorrectly filled in questions were left out for the calculation of the T-score.

## Abbreviations

The following abbreviations are used in this manuscript:

ASD	Autism Spectrum Disorder
DSM-V	Diagnostic and Statistical Manual of Mental Disorders, Fifth Edition
OCD	Obsessive-Compulsive Disorder
GAD	Generalized Anxiety Disorder
CBT	Cognitive Behavioural Therapy
NICE	National Institute for Health and Care Excellence
GI	Guided Imagery
QOL	quality of life
ADHD	attention deficit hyperactivity disorder
MSE	Multi-Sensory Environment
SCAS	Spence’s Children Anxiety Scale
SCAS-P	Spence’s Children Anxiety Scale for Parents

## Appendix A. Technological Choices

This section will highlight some of the technological choices that were made during the design and implementation process. First, in Appendix A.1, the decision process for the game engine will be explained, followed by an in-depth discussion of how procedural generation can ensure some of the quality attributes in Appendix A.2. Finally, Appendix A.3 provides an example of the data object, containing user and gaming data, stored on the server.

### Appendix A.1. Unity

The game engine Unity [80] was chosen as it is an established engine with huge growth in popularity over the past few years. It is used for a variety of games on different platforms. Some well-known games using the Unity game engine are Temple Run [81], Ori and the Blind Forest [82], Monument Valley [83] and Pillars of Eternity [84]. Unity has a personal edition which is freely available to anyone. Games can be commercialized under this persona license, as long as company profits are less than $100,000 annually. Using Unity for the development of the game requires thus no licenses. Furthermore, Unity offers various tutorials and is generally well documented. The community is well established and contains both amateurs and professions. Finally, the Unity game engine also provides the option to develop cross-platform. The initial focus is on the Android platform, both New Horizon and SpaceControl are currently deployable on Android devices, but Unity should allow for easy porting to iOS, web player or other platforms.

### Appendix A.2. Procedural Generation

Procedural content generation is the automatic creation of game content using algorithms with limited or indirect user input [85]. Procedural generation has many purposes, it can be used to promote replayability or to achieve data compression: algorithms can be written that generate new and unique content each time they are executed with different input values, but procedural generation also reduces the amount of data that needs to be stored, opposed to generating content beforehand and then storing it [86]. A well-known example of a game that uses procedural generation is Minecraft [87].

An important aspect of procedural generation is that it should not be random. If content would be generated by placing design elements more or less uniformly on a playing area without any regard to structure, the resulting level would be unplayable. All content generators follow some rules to ensure playability, e.g., the presence of a path from the start to the end of the level [85]. The content generators can be interpreted as random as they include some stochasticity: the content must adhere to strong constraints but, within the constraints, content can vary according to some pseudo-random process [85].

Procedural generation can be used in different ways in game design, which impacts user experience. It can be used to improve the replayability of a game: the gaming experience of the user is different for every playthrough. Related to replayability is adaptability. Adaptability refers to the use of procedural generation to adjust content in reaction to the user’s actions or skill level in the game. Including adaptability in a game can improve replayability [86]. Procedural generation can also be used to augment a game’s dynamics or mechanics. For example, in a first-person shooter game, procedural generation can be used to automatically create a gigantic amount of weapons. The mechanics of the game, first-person shooter, will remain the same, but the dynamics change as the player can constantly change their gameplay to take advantage of a new weapon [86].

Multiple aspects of the game are procedurally generated to create a game with an infinite amount of mini-games while using a limited amount of storage.

Procedural generation is used for the generation of the universe and the mini-games. For the universe, it is used to determine the position, size and types of mini-game of the planets. Since the universe is infinite, it is not generated in its entirety. Only the part of the universe visible for the player will be generated. The mini-games are generated based on the planet’s location and the player’s chosen difficulty level, meaning that a planet with the same mini-game type, but a different location or changing the difficulty level will result in another mini-game of the same type. For example, a different value as input, here the planet’s coordinates, will result in different sequences of stars in the memory mini-game. Only the input value, the planet coordinates, needs to be stored, thus saving space.

To reduce the amount of needed storage space even further, procedural generation can be applied to the artwork and textures as well. During the initial implementation stages, a prototype was made trying to generate the universe background, using textures and procedural generation. This turned out to not be an easy feat since Unity appeared to not be fit for creating a game that uses real-time procedural generation. Executing code in separate threads is necessary for real-time procedural generation as these calculations take time, often longer than the duration of one frame. Since Unity is in charge of the game loop and chooses to execute mostly everything in the main thread, major framerate drops were experienced when trying to generate the background texture using procedural generation. For the proof of concept of New Horizon, described in this paper, all the artwork is created manually.

### Appendix A.3. Data Object

When the user opens the SpaceControl app, an HTTP request is sent to the server to retrieve the user’s data. The data is sent from the server in a JSON object and will contain all the gaming data of the specific user. Listing A1 shows an example of such a JSON object. During a gaming session, the data previously mentioned is collected in the New Horizon game. When the user closes the app, a JSON object is created and sent to the server. If the user wants to view his gaming behavior in the SpaceControl app, the JSON objects corresponding to his user ID will be retrieved. Each JSON object corresponds with the data that has been collected during one day, if daily synchronization took place. Listing A1 shows the data of the user with user ID “SC01” and name “Stéphanie” on 23 May, she has had one gaming session during which she played one mini-game of type 2, the senses mini-game, and indicated a mood of 4, which corresponds with “happy”.

A Wi-Fi connection is needed each time the SpaceControl app is consulted, since the gaming data is only available during a session. The user IDs are stored locally.

**Listing A1.** Example of a JSON object containing game data from a specific user.

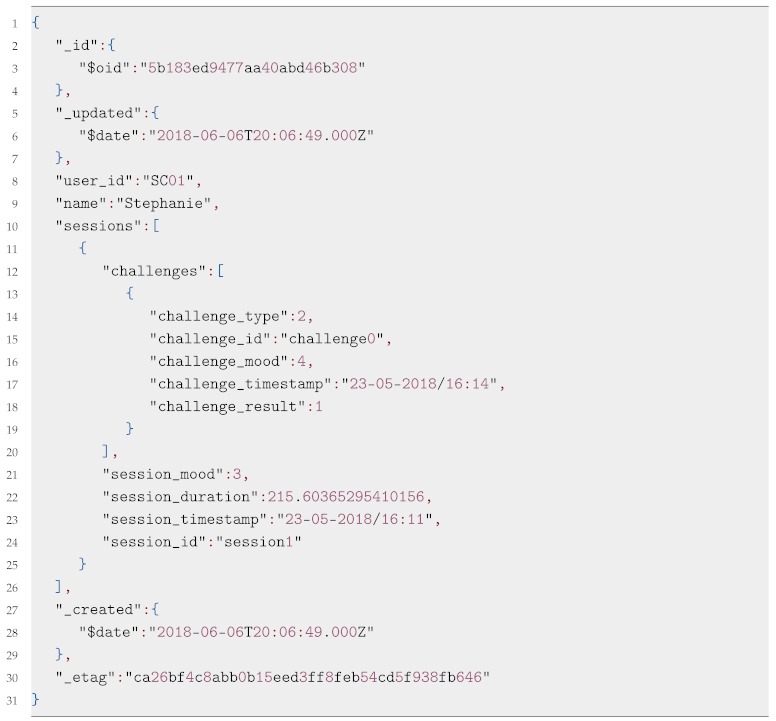

## Appendix B. Implementation

This section will further elaborate on some of the implementation details of the SpaceControl application, in Appendix B.1, and the New Horizon application, more specifically, the two mini-games that do not contain any relaxation techniques, the memory and the platformer mini-game in Appendix B.2 and Appendix B.3 respectively.

### Appendix B.1. SpaceControl: Android Activities

The first version of the SpaceControl application has been kept quite simple, containing only 6 Android activities, namely two related to user actions, i.e., displaying a list of all added users and adding a new user, and 6 activities for information graphs and the home screen, as shown in Figure 3.

- Home: The home screen provides some basic information about the gaming behavior of the child, such as the total time played, average time played per day and average number of gaming sessions per day. It is the first screen the user sees when starting the app. At startup, an HTTP request is sent to the server to retrieve the user’s gaming data. If it is the first time the app is started, a new user will first need to be added, before any data can be retrieved. If the HTTP request could not be completed, an error message will be displayed on the home screen If the data object has been successfully retrieved, it will be converted to data sets, necessary to display the following graphs.
- Mini-game rates: the first graph is a pie chart, indicating the total amount each mini-game has been played. The graph can learn the user what the favorite mini-game of the child is, but also if they are constantly playing the same mini-game over and over again or actually playing different mini-games.
- Mood rates: the second graph is a radar chart. After playing a mini-game, the child needs to indicate on a Likert scale how he/she is feeling. This radar chart displays the relationship between the type of mini-game and the indicated mood of the child. This chart can learn the user if the child often indicates the same mood after playing a certain type of mini-game or if he/she finds a certain mini-game enjoyable or not. Combined with the previous graph, the user might notice that the most played mini-games might not be related to positive feelings, but might elicit rather negative feelings, making the user frustrated while playing.
- Daily view: the third and last graph shows how often and when the sessions took place on a specific date. It also portrays the evolution in the child’s mood for each gaming session. The user can learn from this graph if the child has the tendency to play the game at a fixed moment and if he/she starts to play the game when feeling down. By zooming in on a gaming session, it can be seen if the mood of the child did improve during a gaming session or not.
- New user: to add a new user, a user ID has to be entered. For the user tests of the proof of concept, user IDs have been provided. If the user cannot be found, an error message is displayed.
- User list: multiple users can be added to the app, the user list activity provides an overview of all added users.

### Appendix B.2. Memory Mini Game

The objective of the mini-game is to repeat the sequence by selecting the correct stars in the correct order. During each memory mini-game, the player has to complete two or more sequences of a given length. If the player completes a sequence, the next sequence is shown. Once all sequences have been completed, the mini-game is won. A score is given based on how many mistakes were made and how much time was left. The player starts the mini-game with a set number of chances, depending on the difficulty, as well as with a countdown timer. Should the player run out of chances or time, before all sequences have been completed, the mini-game is lost.

### Appendix B.3. Platformer Mini Game

During a platformer mini-game, the player has to try and reach the spaceship without dying. The level is filled with obstacles, hazards and enemies which will hinder the player’s progress. The reward the player gets is based on the time taken and the lives left. The faster you can complete the mini-game, the higher the reward. Should the player run out of lives before he manages to get to the end of the level, the mini-game is lost.
